# Caecal microbiota composition of experimental inbred MHC-B lines infected with IBV differs according to genetics and vaccination

**DOI:** 10.1038/s41598-022-13512-7

**Published:** 2022-06-15

**Authors:** Marion Borey, Bertrand Bed’Hom, Nicolas Bruneau, Jordi Estellé, Frederik Larsen, Fany Blanc, Marie-Hélène Pinard-van der Laan, Tina Dalgaard, Fanny Calenge

**Affiliations:** 1 INRAE, AgroParisTech, UMR GABI, Université Paris-Saclay, Jouy-en-Josas, France; 2 Institut de Systématique, Evolution, Biodiversité (ISYEB), Muséum National d’Histoire Naturelle, CNRS, Sorbonne Université, EPHE, Université Des Antilles, 75005 Paris, France; 3 Department of Animal Science, Aarhus University, Blichers allé 20, 8830 Tjele, Denmark

**Keywords:** Adaptive immunity, Infection, Live attenuated vaccines, Animal breeding, Microbial genetics, Next-generation sequencing

## Abstract

Interactions between the gut microbiota and the immune system may be involved in vaccine and infection responses. In the present study, we studied the interactions between caecal microbiota composition and parameters describing the immune response in six experimental inbred chicken lines harboring different MHC haplotypes. Animals were challenge-infected with the infectious bronchitis virus (IBV), and half of them were previously vaccinated against this pathogen. We explored to what extent the gut microbiota composition and the genetic line could be related to the immune response, evaluated through flow cytometry. To do so, we characterized the caecal bacterial communities with a 16S rRNA gene amplicon sequencing approach performed one week after the IBV infectious challenge. We observed significant effects of both the vaccination and the genetic line on the microbiota after the challenge infection with IBV, with a lower bacterial richness in vaccinated chickens. We also observed dissimilar caecal community profiles among the different lines, and between the vaccinated and non-vaccinated animals. The effect of vaccination was similar in all the lines, with a reduced abundance of OTU from the *Ruminococcacea* UCG-014 and *Faecalibacterium* genera, and an increased abundance of OTU from the *Eisenbergiella* genus. The main association between the caecal microbiota and the immune phenotypes involved TCR_ϒδ_ expression on TCR_ϒδ_^+^ T cells. This phenotype was negatively associated with OTU from the *Escherichia*-*Shigella* genus that were also less abundant in the lines with the highest responses to the vaccine. We proved that the caecal microbiota composition is associated with the IBV vaccine response level in inbred chicken lines, and that the TCR_ϒδ_^+^ T cells (judged by TCR_ϒδ_ expression) may be an important component involved in this interaction, especially with bacteria from the *Escherichia*-*Shigella* genus. We hypothesized that bacteria from the *Escherichia-Shigella* genus increased the systemic level of bacterial lipid antigens, which subsequently mitigated poultry γδ T cells.

## Introduction

Microbes are becoming increasingly resistant to the antibiotics used to treat human and animal infections. This is why the use of antibiotics as growth promoters (AGP) in animal production is forbidden in a growing number of countries. In chicken flocks, the ban of antibiotics has been followed by the observation of more frequent gastro-intestinal disorders such as necrotic enteritis^1^. Moreover, genetic selection on production traits such as growth rate, feed efficiency or meat and carcass yield may have indirectly impacted animal robustness, since immune phenotypes or disease resistance phenotypes were generally not included in selection programs by breeders^2,3^. For instance in broiler breeding, selection for an increased body weight led to a decrease in the relative weight of primary and secondary immune organs^4^. Intense selection may also have compromised the host immune response to *Escherichia coli* vaccination^5^ or increased the susceptibility to Marek’s disease^6,7^. Potential trade-offs between productivity and immunity are often hypothesized to explain such results^8^.

In order to avoid economic losses due to infections by pathogens and to ensure animal health in the absence of antibiotics, reinforced vaccination and the inclusion of probiotics in animal feeding were among the first approaches considered^9^. In this context, increasing the efficiency of vaccines may also be considered. Vaccines are still suboptimal for many endemic chicken diseases due to the variety of geographical areas, types of production, and poultry species and breeds, which all influence the variability of response to the vaccine and the outcome of the infection^10–12^. Most of efficiency evaluations of experimental vaccines comprise measurements of the immunological response and disease outcome after submitting vaccinated animal to a suitable challenge mimicking natural infection^13^.

Several approaches have been considered to enhance the animal immune response to vaccination, and some may depend on the intestinal microbiota^14,15^. Certain strategies focus on the early manipulation of intestinal innate lymphoid cells (ILCs)^16^. Besides, many new vaccine designs specifically target the antigen-presenting cells (APC) and use bacterial components as adjuvants through ligand-based APC targeting. For instance, TLR ligands have been used as adjuvant in chickens immunized with inactivated avian influenza IAV H5N2 co-administrated with *Salmonella enterica* flagellin^11,17^, and an enterobactin conjugate vaccine demonstrated potent ability to elicit high antibody response against *Campylobacter jejuni* in chicken^18^.

In parallel, the early modulation of the intestinal microbiota is already promoted to improve intestinal health of chickens^19,20^. Studies report the effects of microbiota modulatory compounds such as probiotics: *Anaerosporobacter mobilis* or *Lactobacillus reuteri* impact the outcome of vaccination against *Campylobacter jejuni*^21,22^. Moreover, variations in the commensal gut microbiota composition can affect adaptive immune responses in vaccinated chickens, with higher hemagglutination titers in chicken submitted to a probiotic treatment based on several *Lactobacillus* species in comparison with antibiotic treated chicken^23^. A few recent studies report also the effect of vaccination on the gut microbiota, for instance against *Salmonella*^24^. In addition, vaccines with attenuated *Eimeria* have been shown to diminish the microbial diversity and *Firmicutes* relative abundance in broilers^25,26^. The interactions between vaccination and the gut microbiota remain to be studied for a larger set of pathogens, and for different types of vaccines.

In addition to the intestinal microbiota, animal genetics is a parameter scarcely considered when developing vaccination strategies. Adjusting vaccines to the host genetics, or genetically selecting animals displaying a better immune response to vaccines, could be a relevant way of improving the vaccine response in farm conditions. For example, in chickens, strong associations occur between many infectious diseases and the genotype at the MHC (Major Histocompatibility Complex), which is a polymorphic region comprising genes coding for cell surface molecules involved in antigen presentation^27^. Genetic variations at the MHC might also influence the vaccine responses, as observed for instance for the Newcastle disease vaccine^24^ and IBV^28,29^.

The Infectious Bronchitis Virus (IBV) is a single-stranded RNA and enveloped Coronavirus causing a respiratory disease in infected chickens. The high rate of mutations of this virus is leading to the existence of many strains with different virulence levels. The morbidity through respiratory symptoms in infected husbandry is high. Despite vaccination schedules, chickens still display suboptimal levels of protection worldwide. In layer hens, IBV leads to economic losses with impaired egg production and quality. Typically, the egg laying declines observed range between 3 and 10%, but reductions of up to 50% have also been observed^30^.

Based on flow cytometry assessments of peripheral blood cells among experimental inbred MHC-B lines infected with IBV, Larsen et al. reported that the animals with the highest monocyte MHC-II expression had the weakest vaccine-induced challenge protection^29^. We used the animals from this study to investigate if their caecal microbiota was associated with their immune phenotype profiles. Vaccinated challenged animals displayed a mild infection with a reduced tracheal IBV carriage, in comparison to non-vaccinated challenged animals that displayed an acute infection with higher tracheal IBV carriage. Furthermore, in vaccinated animals, different susceptibility levels to the challenge infection were observed between MHC lines, with chickens of the MHC haplotype B21 displaying low IBV loads after challenge, and thus the best responses to the IBV vaccination.

In the present study, we wondered if the vaccination and the IBV challenge of these six chicken MHC lines were also associated with differences in caecal microbiota composition. To investigate it, we compared vaccinated and non-vaccinated animals to identify associations present during either a sub-clinical infection, or an acute infection. We explored if the caecal microbiota of the MHC lines differed one week after IBV challenge and assessed whether the variations of various immune parameters could be associated with the caecal microbiota composition.

## Results

**Figure 1:**
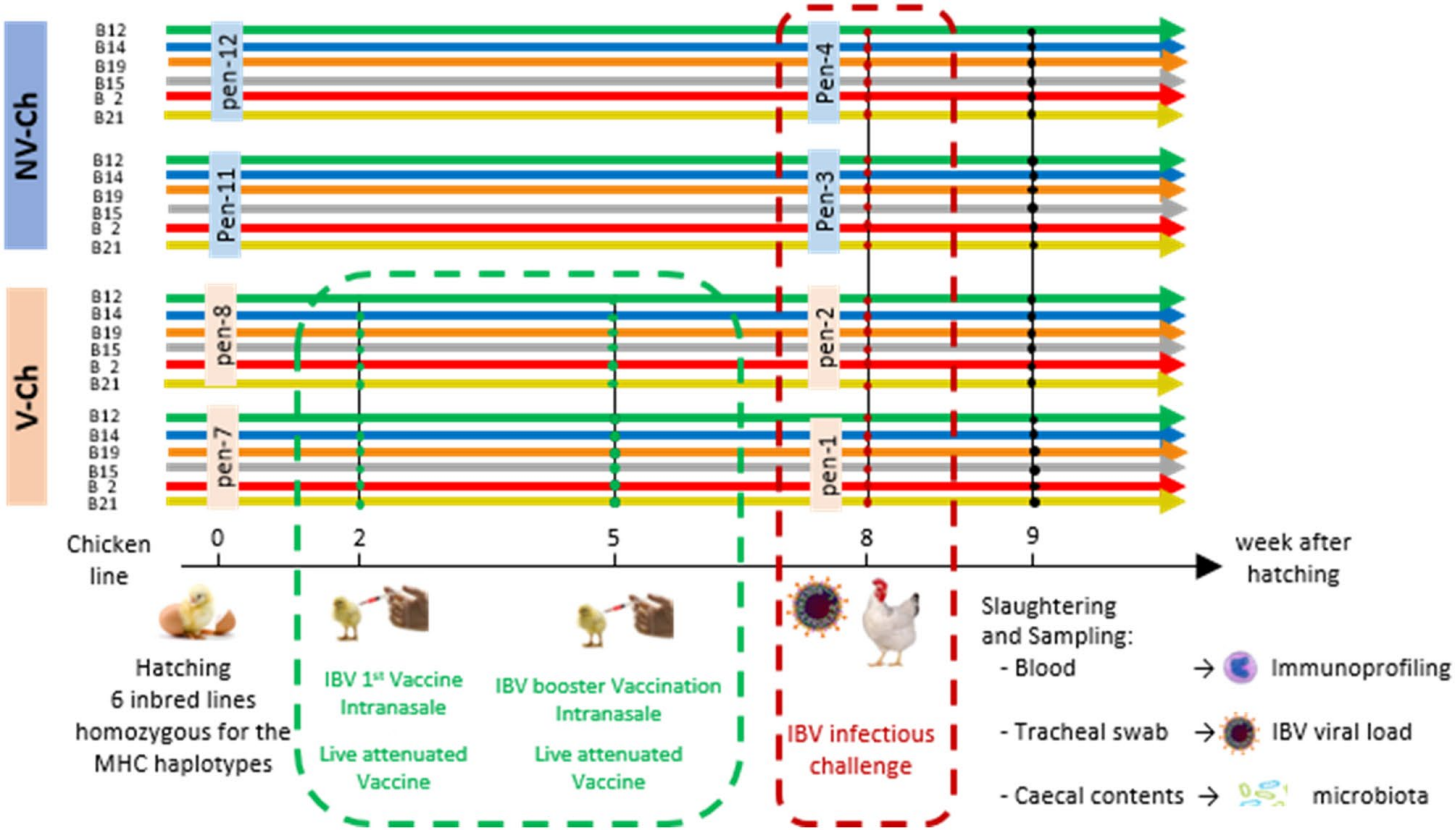
Experimental design and IBV load after challenge. A group of 48 vaccinated and challenged (V-Ch, n_B12_ = 8, n_B14_ = 8, n_B19_ = 8, n_B15_ = 8, n_B2_ = 8, n_B21_ = 8) individuals was opposed to a group of 45 non-vaccinated and challenged (NV-Ch, n_B12_ = 6, n_B14_ = 7, n_B19_ = 8, n_B15_ = 8, n_B2_ = 8, n_B21_ = 8) individuals, with 6 lines of White Leghorn laying hens followed in each group. Two pens per vaccine status were used, and the lines were reared together.

In the present study, we studied the interactions between caecal microbiota composition and parameters describing the immune response in six experimental inbred chicken lines harboring different MHC haplotypes. The chicken lines were challenge-infected with the infectious bronchitis virus (IBV), and half of them were previously vaccinated against this pathogen (Fig. 1). We explored to what extent the gut microbiota composition and the genetic line could be related to the variability of the immune response, evaluated through flow cytometry. To do so, we characterized the caecal bacterial communities with a 16S rRNA gene amplicon sequencing approach performed one week after the IBV infectious challenge. In an associated study on the same animals, Larsen previously categorized the 6 chicken lines according to the levels of their tracheal IBV load 5 days post infection in the vaccinated and non-vaccinated group to assess the capacity of the chicken to dampen the infection^29^. B21 animals that displayed the lowest viral loads after challenge in the V-Ch group, and had the highest load in the NV-Ch group were thus qualified as susceptible to IBV infection and high responder to vaccination.

### Global taxonomic composition of caecal microbiota after IBV challenge in vaccinated and non-vaccinated chickens

**Figure 2:**
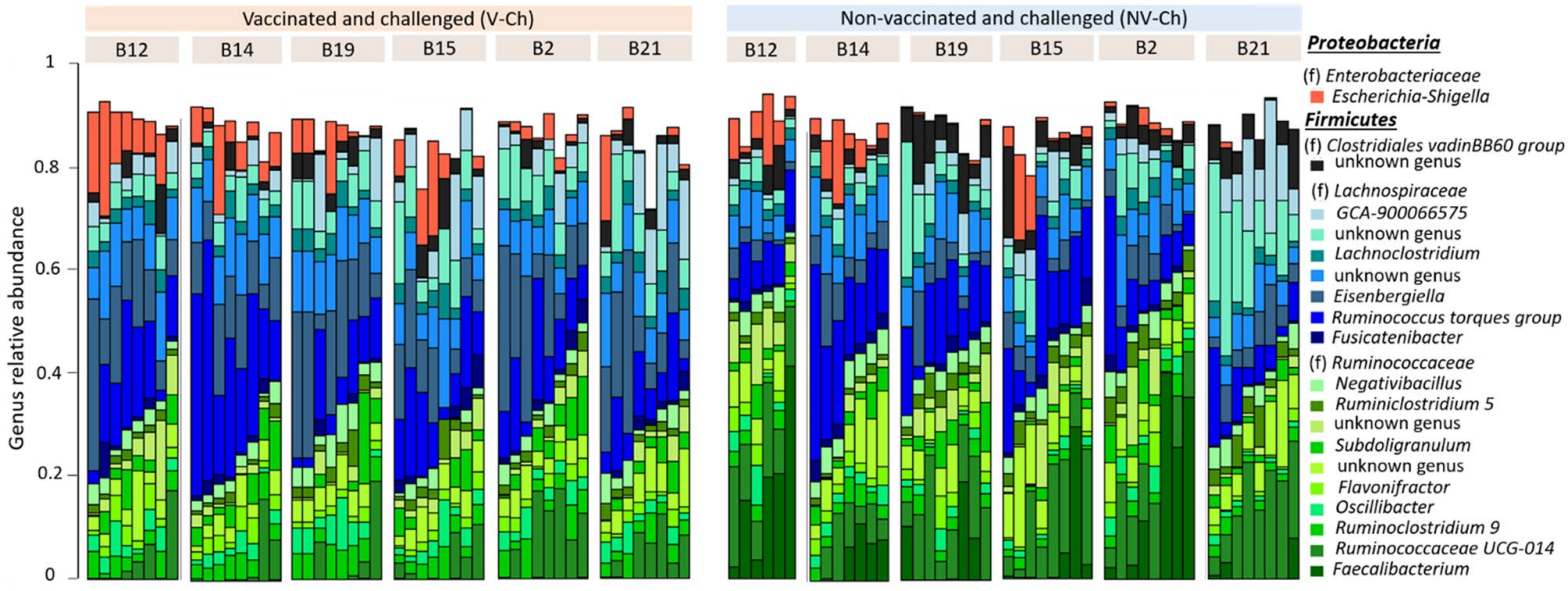
Caecal microbial community of 9-week-old layers, one week after IBV infection. Taxonomic composition is given at the genus level among the different lines, and according to their vaccine status: vaccinated and challenged (V-Ch, n = 48) vs non-vaccinated and challenged (NV-Ch, n = 45). Every bar corresponds to one individual. Only taxons with a mean relative abundance above 2% are displayed.

From the total population composed of 93 caecal samples from V-Ch and NV-Ch groups, 16S rRNA gene sequencing provided a total of 12,256,442 reads, reduced to 4,952,178 sequences after quality control with an average of 53,249 sequences per sample (range from 32,560 to 76,819 sequences). We identified 487 OTU from 5 phyla, 12 families and 73 genera (Fig. 2). The massively dominant phylum was *Firmicutes* (average abundance 96%), with essentially *Lachnospiraceae* (48.1%), *Ruminococcaceae* (42.1%), and to a lesser extent *Bacilli* (2.1%). It was followed by the much less abundant *Proteobacteria* (average abundance 3.9%) (Supplementary Table S1; Supplementary Fig. S1). Over the 73 genera identified, *Ruminococcus torques* group (12.4%), *Eisenbergiella* (9.0%), and *Ruminococcaceae UCG-014* (8.0%) were the three dominant ones, and 34 significant correlations were observed between their abundances. *Eisenbergiella* was negatively associated with *Ruminococcaceae UCG-014* (ρ = -0.52), and *Escherichia-Shigella*, which is often described as pathogenic, was positively associated with *Anaerostipes* (ρ = 0.54), and with an unknown genus from the *Gammaproteobacteria* class (ρ = 0.95) (Supplementary Table S2, Supplementary Fig. S2).

### Effect of vaccination on the caecal microbiota after IBV challenge

In vaccinated animals, one week after the IBV challenge, species richness (p = 7.0 × 10^–4^) and Chao1 estimator (p = 7.2 × 10^–4^) were reduced in caecal microbiota composition when compared to non-vaccinated animals. But no significant effects, neither on Shannon (p = 0.24) nor on inverse Simpson indexes (p = 0.92), were found (Table 1). As *Lachnospiraceae* and *Ruminococcaceae* were the dominant families observed, we wondered whether vaccination had also an effect on species Richness and Chao1 in those two families separately. In the *Ruminococcaceae* subgroup, vaccination significantly decreased OTU richness (p = 1.1 × 10^–7^) and Chao1 (p = 9.8 × 10^–7^), but it had no significant effect in the *Lachnospiracea* group (Supplementary Fig. S3).

**Table 1:** Effect of IBV vaccination on the α-diversity of the caecal microbial community of 9-week-old hens, one week after their infection with IBV.

OTU set (number)		V-Ch		NV-Ch		P value		FDR
All OTU (487)	Richness	320		351		7.0 × 10^–4^		0.005*
	Chao1	342		374		7.2 × 10^–4^		0.006*
	Shannon	3.85		3.93		0.24		0.416
	InvSimpson	22.8		23.2		0.92		0.960
OTU from the *Ruminococcaceae* family (217 OTU)	Richness	131		157		1.1 × 10^–7^		**10**^**–6**^ ***
	Chao1	140		165		9.8 × 10^–7^		**3.10**^–6^***
OTU from the *Lachnospiraceae* family (219 OTU)	Richness	159		157		0.66		0.907
	Chao1	170		170		0.98		0.960

α-diversity indices among the vaccinated and challenged (V-Ch) and the non-vaccinated and challenged (NV-Ch) groups, with the richness index representing the number of observed OTU. The vaccination effect is first described in the total bacterial community (first line ‘All OTU’) and then within the *Ruminococcaceae* (second line) and *Lachnospiraceae* (third line) families separately. We indicated the mean of each alpha-diversity indices in the V-Ch NV-Ch groups, and set the FDR of the vaccination effect in bold letter when significant (*< 0.05, and ***< 0.001). Alpha diversity indices are available in Supplementary Table S5.

**Figure 3:**
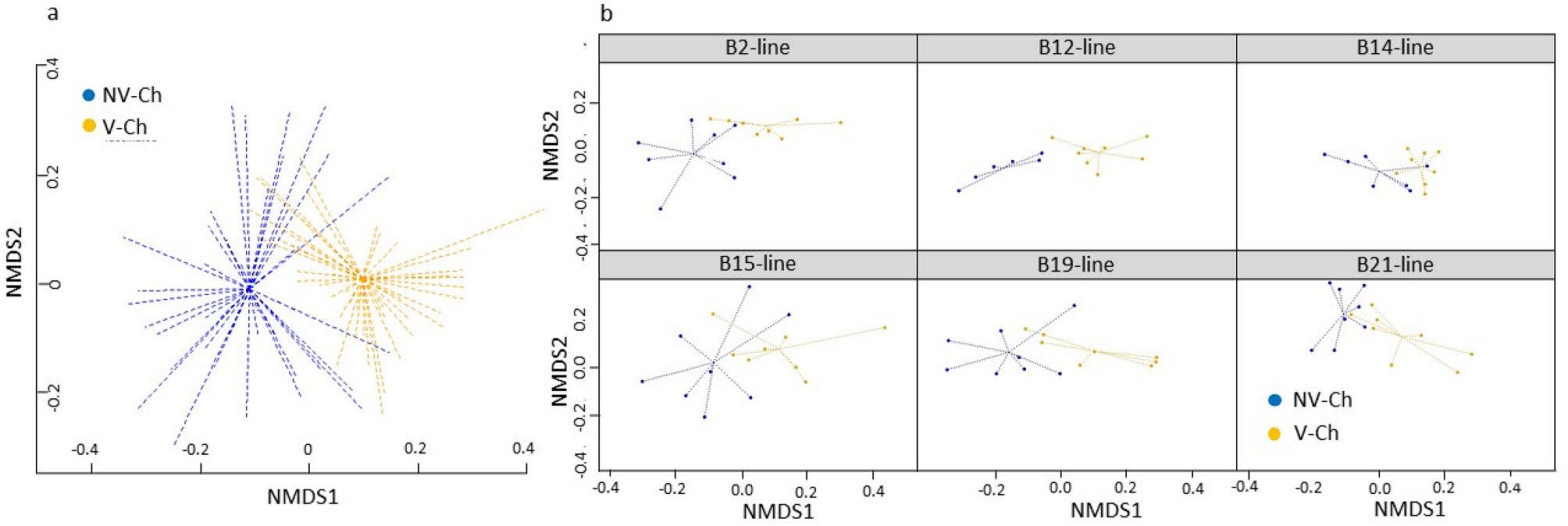
Effect of IBV vaccination on the caecal microbial community of 9-weeks-old hens infected with IBV. (**a**) NMDS representation of the caecal microbial communities using Bray–Curtis distances among the vaccinated challenged (V-Ch, n = 48) (orange) and the non-vaccinated challenged (NV-Ch, n = 45) (blue) groups considering all the lines together. (**b**) NMDS representation of the caecal microbial communities in each MHC line separately. Arrows represent the centroids displacement from the non-vaccinated challenged (NV-Ch) group (blue) to the vaccinated challenged (V-Ch) group (orange).

Regarding β-diversity, vaccination had a significant effect on Bray–Curtis distances (p = 1 × 10^–4^) with V-Ch animals forming a distinct cluster from the NV-Ch animals on NMDS representation (Fig. 3a). Furthermore, it strengthened microbiota homogeneity as it significantly reduced β-diversity (p = 4 × 10^–3^). Finally, by looking at the centroids displacement on an NMDS from the NV-Ch group to the V-Ch group, we observed that the microbiota was similarly modified with the vaccination in all lines (Fig. 3b).

Differential analysis on OTU abundance between the V-Ch and NV-Ch groups showed that among the differentially abundant OTU, 23 out of 25 from the *Ruminococcaceae UGG-014 group* genus, and 5 out of 6 OTU from *Faecalibacterium* genus were less abundant in the vaccinated group. On the contrary, the following components were more abundant in the vaccinated group: most OTU from the *Eisenbergiella* genus (11 out of 12), the *Ruminococcus torques group* (5 out of 7), the *Ruminoclostridium 9* group ; the *Oscillibacter* genera (5 out of 6); *Escherichia-Shigella*, *Lachnospiraceae NK4A136 group, Subdoligranulum,* and *Lactobacillus* genus (3 out of 3); and both OTU from the *Tyzerella* 3 genus (Supplementary Table S3).

Considering the lines separately, the trend for *Ruminococcaceae* was similar for each line, as most of the OTU significantly different in this family were less abundant in the vaccinated group. *Ruminococcaceae UCG-014*, which was the most affected genus within this family, was impacted significantly in the B2, B19, and B21 lines. Finally, the statistical effect of vaccination on OTU abundances was the most important in the B2 line (39 OTU differentially abundant), followed by the B19 line (22 OTU), and to a lesser extent by the B15 and B21 lines (9 OTU) (Supplementary Table S4).

### Differences in microbiota composition according to the genetic line

**Figure 4:**
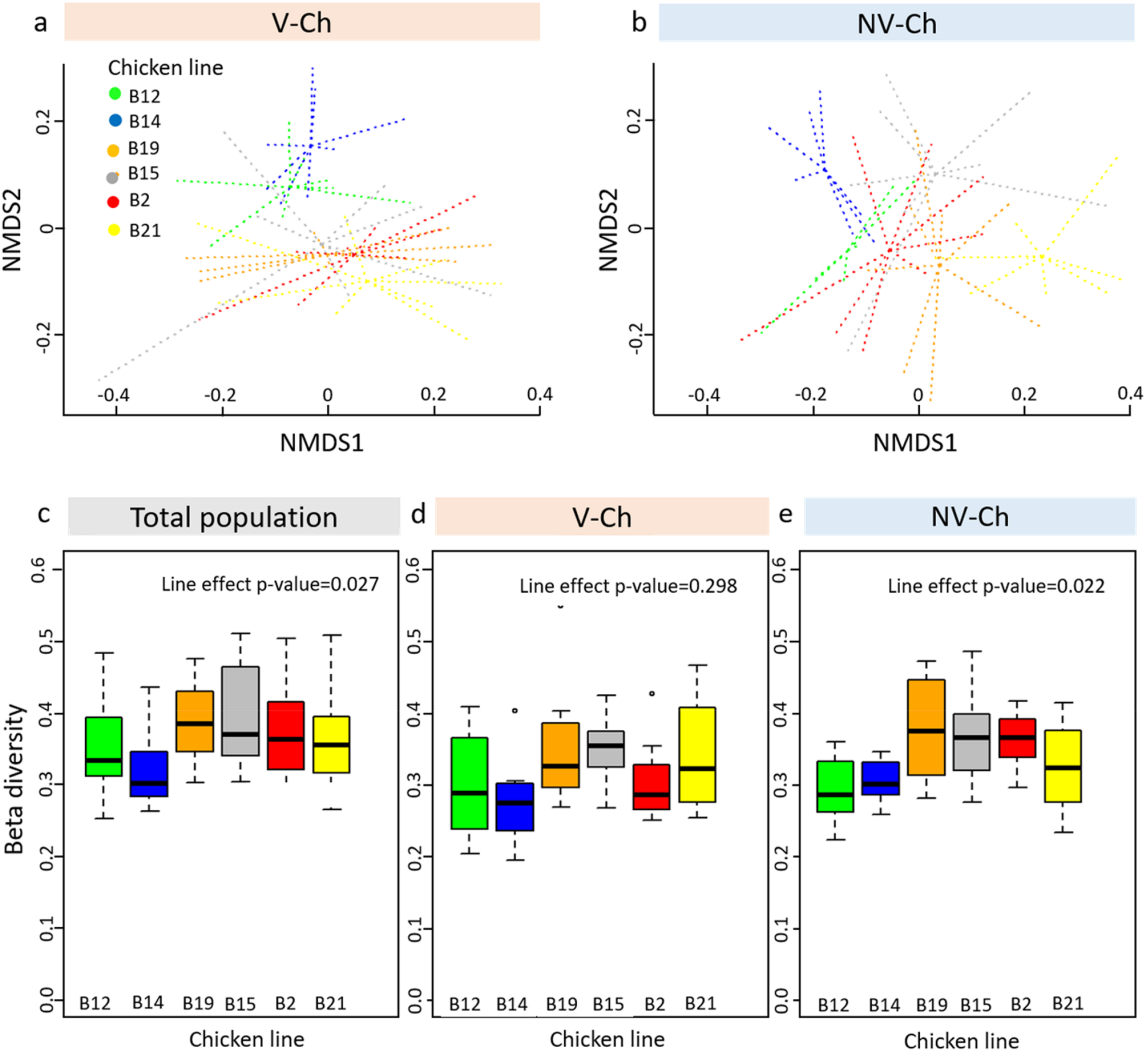
Association between the MHC line and the caecal microbial beta-diversity in laying hens one week after their infection with IBV. NMDS representation of the caecal microbial communities using Bray–Curtis distances among the different MHC lines in the (**a**) vaccinated and challenged (V-Ch, n = 48) and in the (**b**) non-vaccinated and challenged (NV-Ch, n = 45) groups. Representation of the community dispersion in each MHC-line through its β-diversity among (**c**) the total population, (**d**) the vaccinated challenged (V-Ch) group, and (**e**) the non-vaccinated challenged (NV-Ch) group. We indicated the p-value of the line effect. Asterisks indicate a significant difference between two lines based on post-hoc comparison.

Genetic lines were significantly associated with the β-diversity both in the V-Ch and NV-Ch groups (p = 1 × 10^–4^) (Fig. 4a,b), but not with the α-diversity indices (Supplementary Fig. S4, Supplementary Table S5). The microbial dissimilarity observed between the lines was thus not due to a higher bacterial richness in some of them.

Results from pairwise comparisons of BC distances between lines showed that the B14 line displayed both the greatest dissimilarity with the other ones (all pairwise p < 0.0002), and the greatest homogeneity within its bacterial community (Table 2; Fig. 4c–e). The B14 line was followed by the B21 line in terms of dissimilarity with the other lines, and then by B2 and 12 to a lesser extent. Furthermore, we observed a closer proximity between B12 and B14, and between B19 and B21. The number of OTU with significant differential abundance between each couple of lines ranged from none between lines that did not display significant dissimilarity (B12–B15 and B2–B21), to 126 between the B14 and B21 lines that displayed the greatest dissimilarity (Table 2; Supplementary Table S6). Globally, the B14 line had more abundant OTU from the *Ruminococcus torques* group, and lower abundances of OTU from *Ruminococcaceae UCG-014* and *Ruminiclostridium 5* genus (Supplementary Table S7).

**Table 2:** Microbial dissimilarity between each couple of lines of laying hens one week after their infection with IBV.

		B2-line	B12-line	B14-line	B15-line	B19-line
p value	B12-line	0.0026**				
	B14-line	0.0001***	0.0002***			
	B15-line	0.0026**	0.0226*	0.0001***		
	B19-line	0.2002	0.0043**	0.0001***	0.2107	
	B21-line	0.0002***	0.0001***	0.0001***	0.0041**	0.0362*
	B12-line	36				
Number of differentially abundant OTU	B14-line	87	19			
	B15-line	16	0	47		
	B19-line	9	40	74	2	
	B21-line	0	105	126	2	1

Microbial dissimilarity was assessed with Adonis test performed on Bray–Curtis distances between each couple of lines, and the corresponding p-value are reported in the table, and highlighted in bold letter when significant (*< 0.05. **< 0.01. and ***< 0.001). The whole population (vaccinated and challenged V-Ch, and non-vaccinated and challenged NV-Ch) was considered (n = 93). The vaccine status was set as main effect in the Adonis model for the analysis on the beta-diversity, and in the zero-inflated Gaussian mixture model from MetagenomSeq R package for the differential analysis on OTU abundance. Corresponding Supplementary Table S6 provide results of the differential abundance between each couple of lines.

The B21 line is mostly dissimilar from B12 and B14, and it globally harbored a larger abundance of OTU from *Ruminococcaceae UCG-014* and *GCA-900066575* genera and a lower abundance of OTU from *Flavonifractor*, *Escherichia-Shigella,* and *Oscillibacter* genera. Finally, the B12 line harbored a higher abundance of OTU from *Escherichia-Shigella* genus in comparison with the B2, B21 and B19 lines (Supplementary Table S6).

The same pattern was observed considering the V-Ch and NV-Ch groups separately. There was no line effect on α-diversity in any group, and once again, the B14 and B21 lines displayed the most dissimilar microbiota in both groups (Fig. 4a,b; Supplementary Table S6). However, vaccination brought these lines closer: they were less dissimilar in the V-Ch group than in the NV-Ch group, especially for B21 line. All p-values were lower than 0.006 in pairwise comparison on BC distance involving B21 line in the NV-Ch group, while only comparisons between B21 line with B12 and B14 lines displayed significant p-value in the V-Ch group.

As microbiota dissimilarity between the lines could be due to weight differences, we considered lines weight. There were only slight variations in weight between genetic lines, with the exception of line B12, which was on average heavier than other lines, in both the vaccinated group and the non-vaccinated group (Supplementary Fig. S5). It is to notice that the microbiota from the B12 line was not the most dissimilar. Furthermore, there was no significant effect of weight on gut microbiota composition, neither with alpha, nor with beta-diversity. Thus, we conclude that the dissimilarity in microbiota composition we observed between the lines was not due to weight differences.

### Associations between immune phenotypes and microbiota according to the genetic line

Globally, in the total population, using Adonis analysis we identified four immune parameters that shared a significant association with the caecal microbiota from the whole cohort. TCR_ϒδ_ expression on TCR_γδ_ T cells was the immune parameter with the most significant association (p = 5 × 10^–4^). Three other associated immune parameters displayed weaker associations: Bu1 expression on B cells, IgM expression on B cells, and CD45 expression on monocytes (Table 3). The analysis of each line separately showed that for TCR_ϒδ_ expression on TCR_γδ_ T cells, the B21 line displayed the strongest association (p = 0.008), followed by the lines B14 and B15 (p values of 0.02 and 0.04 respectively), while no association was found within B2, B12, and B19 lines. In order to increase the statistical power of our analysis, we performed again this model on a subset of the three lines (B21, B14 and B15) considered together and on a subset of the three other lines (B19, B12, and B2), and represented the results on the total population on an NMDS plot (Supplementary Fig. S6). As expected, TCR_ϒδ_ expression on TCR_γδ_ T cells was significantly associated to the Bray–Curtis distances of the caecal microbiota only in the total population (p = 0.0002) and the B21–B14–B15 sub-group (p = 0.0025). Considering the other immune parameters, the B21 and B19 lines were the only ones that displayed a significant association with Bu1 expression on B cells (respective p-values of 0.03 and 0.01), while the line B19 had an association with serum titer of IBV-specific antibodies (p = 0.02), and the line B21 had an association with CD45 expression on monocytes (p = 0.02).

**Table 3:** Association between the caecal microbiota and immune phenotype in laying hens one week after their infection with IBV.

Immune phenotype	Total population	B12-line	B14-line	B19-line	B15-line	B2-line	B21-line
Monocytes count	0.0652	0.3261	0.3319	0.167	0.7232	0.3555	0.3921
TCRgd-CD4 + CD25 + (ab) T cells count	0.5448	0.7578	0.9323	0.0123*	0.0187*	0.2607	0.7122
CD45 expression on heterophils	0.2911	0.3954	0.7948	0.5906	0.0176*	0.9433	0.4321
CD45 expression on monocytes	0.0303*	0.1889	0.1887	0.099	0.9327	0.075	0.0191*
BU1 expression on B cells	0.0116*	0.2249	0.0754	0.0116*	0.4018	0.3529	0.0272*
IgM expression on B cells	0.0159*	0.0906	0.4423	0.7193	0.8499	0.5344	0.0985
TCR_ϒδ_ expression on TCR_δϒ_ T cells	0.0005***	0.1574	0.0157*	0.6884	0.0354*	0.1957	0.0079**

Analysis of β-diversity on caecal microbiota by Adonis test. Associations between the microbiota and the concomitant immune phenotype are described through the p-value of Adonis test based on Bray–Curtis distance matrix and each immune parameter level. The associations are described in the total population, and in each line separately. When significant, the p-value of the effect is highlighted in bold characters (*p < 0.05, ** < 0.01, *** ≤ 0.001). Supplementary Table S9 provides the complementary results of Adonis model performed on all parameters followed from the immune phenotype, and that did not display significance.

### Associations between immune phenotypes and microbiota composition of chicken with a varying infection severity after an IBV challenge

**Figure 5:**
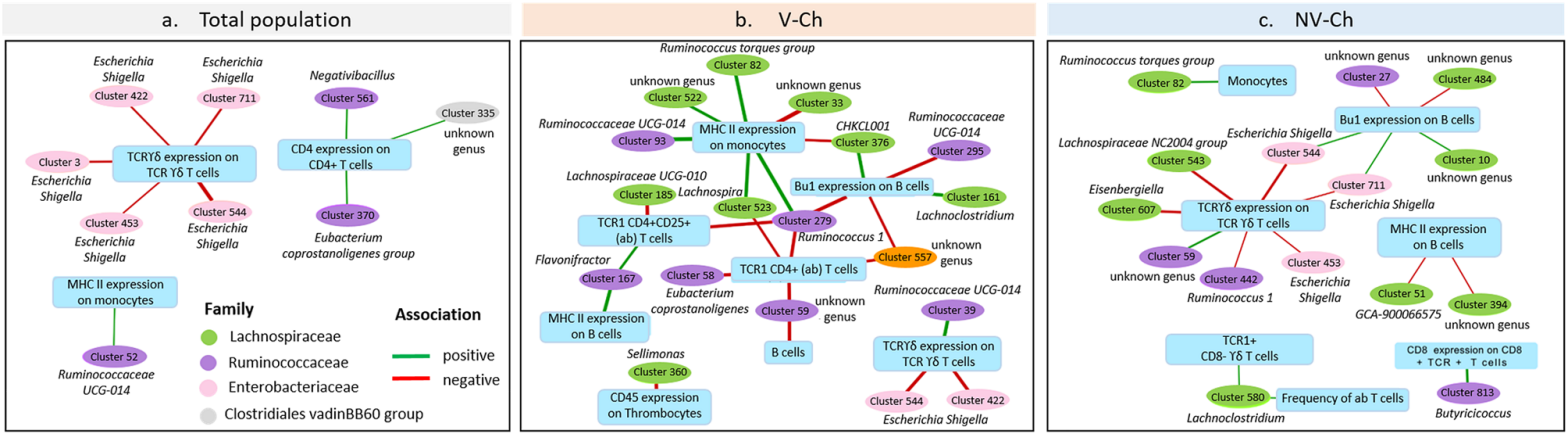
Network representing the associations observed between the immune parameters and the OTU abundances in laying hens, one week after their infection with IBV. Associations were calculated with Spearman correlation in (**a**) the total population, (**b**) the vaccinated and challenged (V-Ch) group, and (c) the non-vaccinated and challenged (NV-Ch) group, with positive associations represented with green segments, and negatives ones with red segments. Only significant (p < 0.05), intermediate (total population: 0.4 < r < 0.6; V-Ch and NV-Ch groups: 0.5 < r < 0.7) and high (r > 0.6, thick segments) correlations are shown.

Finally, we searched for the bacteria that could be involved in these associations through Spearman correlation analysis that we calculated between the level of each immune parameters and the relative abundance of each OTU. We first calculated them on the entire population of vaccinated and non-vaccinated birds to search for associations present whatever the level of IBV infection (Fig. 5a; Supplementary Table S10). Then, we calculated it separately on the animal from the V-Ch group (Fig. 5b; Supplementary Table S11) and from the NV-Ch group (Fig. 5c; Supplementary Table S12) to identify associations specific of an acute infection with an inflammatory state group (NV-Ch) or of a rather sub-clinical infection (V-Ch).

We only got few common associations between these three analyses. We observed negative associations between TCR_ϒδ_ expression on TCR_γδ_ T cells and OTU from *Escherichia-Shigella* genus in the three datasets. Especially, Cluster 544 from this genus was observed in the three datasets, whatever the level of infection.

All the other associations we observed in the V-Ch group and the NV-Ch group implied quite different immune parameters and different OTU. The highest correlations were in the V-Ch group with, for instance, two strong positive correlations implying MHC-II expression on monocytes: one with Cluster 93 from *Ruminoccocaceae UGC-014* genus (ρ_C_ = 0.63, adjusted-p = 0.001), and one with Cluster 279 from *Ruminococcus 1* genus (ρ = 0.62, adjusted-p = 0.001).

## Discussion

In the present study, we wondered if the vaccination and the IBV challenge of six chicken MHC lines were associated with differences in caecal microbiota composition. We report the observation of statistical associations between both the vaccination to IBV and the genetic line, and the caecal microbiota in a context of IBV infection. Animals were studied one week after an intranasal viral infection. This experimental IBV challenge enabled us to fully follow the individual capacity of birds to decrease the viral load and thus their level of protection and recovery, as serum IBV-specific immunoglobulin level per se is not a good predictor of vaccine-induced protective immunity^31,32^. Based on the tracheal IBV load after experimental infection, the B21 line appeared to show the most efficient response to IBV vaccination. We wondered whether the caecal microbiota could also display associations with the cellular immune profiles in peripheral blood. We first identified significant associations between the vaccine status (vaccinated challenged vs non vaccinated challenged) and the caecal microbiota composition on the one hand, and between the genetic line and the caecal microbiota on the other hand. We then investigated the associations between the caecal microbiota composition and immune phenotypes of the challenged animals in the total population and in the V-Ch and NV-Ch groups separately. A recent study reported the effect of nephropathogenic IBV (NIBV) infection on the ileal microbiota of Hy-Line brown chickens^33^. It was observed that NIBV disturbed the intestinal epithelium, with an hyperplasia of goblet cells, and a lowered ratio of the villus height to crypt depth. NIBV also disrupted the ileal microbiota with a reduction in the OTU richness, *Firmicutes* and *Cyanobacteria* phyla and of *E.coli*, and with an enrichment of phylum *Proteobacteria* and *Bacteroidetes.* Based on those results, it was hypothesized that the intestinal inflammation should have increased the availability of oxygen, which must have favored *Proteobacteria* bloom and intestinal dysbiosis. Although a direct comparison of our results with this study is not very relevant since the experimental design differs from our design in many ways, such results confirm our hypothesis that the gut microbiota composition may actually interact with the host response to IBV.

### Effect of vaccination on caecal microbiota composition after IBV challenge

In the present study, the bacterial richness and the Chao1 estimator were lower in V-Ch animals, as compared to NV-Ch animals, especially in the *Ruminococcaceae* family. As we did not observe a significant effect on the Shannon index that is more sensitive to the more abundant OTU, we can hypothesize that the greater richness from the NV-Ch group could be due to its higher carriage of low abundant OTU. Our results are in accordance with other reports on viral infections caused by *Influenza* or the Respiratory Syncytial Virus (RSV)^34,35^. We also noticed that NV-Ch and V-Ch groups had a dissimilar microbiota, with differences observed on the OTU relative abundances between the two groups that were similar in all the lines. Globally, the vaccinated and challenged animals displayed a significant reduction of the abundance of OTU from the *Ruminococcacea* UCG-014 and *Faecalibacterium* genera, and a significant increase of the relative abundance of OTU from the *Eisenbergiella* genus, and to a lesser extent of *Ruminococcus* torques group, *Tyzzerella* 3, and *Ruminoclostridium* 9 or 5 genera. Due to the IBV viral challenge, we can assume that the vaccination effect we observed was mostly the consequence of a different level of infection between the unprotected control and vaccinated groups. Thus, we cannot exclude that the differences we observed on the caecal microbiota between the control and the vaccinated group may have resulted from the differences of those clinical states. There are only a few reports on the effect of vaccination on the intestinal microbiota, with observations made on uninfected individuals, and the results are contradictory^34,36,37^. Investigating the effect of vaccination on gut microbiota in a context of infection is thus of interest to improve vaccine design. Beside vaccination and animal clinical state, another possible source of microbiota variation that cannot be excluded is a possible difference in the environmental microbiota to which animals were exposed, since vaccinated and non-vaccinated animals had to be reared into two different units.

### An association between genetic lines and caecal microbiota composition

We observed that the microbial communities of the six genetic lines were dissimilar in their global composition, as assessed by the comparisons of their Bray–Curtis distances and the differential analysis performed on their OTU abundances. In our experimental design, animals from the different lines were reared in the same pens and were thus pecking a litter containing faeces from all the lines^38,39^. The differences observed between lines therefore do not come from a different exposure to environmental microbes, thus strengthening the relevance of the association we observed between the host genetic background and the microbiota. Intestinal microbial dissimilarities have been reported among different poultry breeds and different broiler lines, for instance between lines genetically divergent for their digestive efficiency, or differing for their susceptibility to *Salmonella* Enteritidis and *Campylobacter jejuni*^40,41^. These microbial dissimilarities between lines of animals reared in the same environment could have resulted from physiological particularities related to their immune system^42^, or from differences in their digestive anatomy^43–46^. In the present study, the differences observed between lines could be due to genetic variations in the MHC locus but not only: the lines we studied are not isogenic. They may differ genetically for genomic regions other than the MHC locus, which could also have an effect on the caecal microbiota composition. Progenitors of the studied animals were reared in the same environment, so that we can discard the hypothesis made in other studies of a selecting pressure exerted by the environment through yolk antibodies^38,47^. This reinforces our hypothesis that genetic differences shaped the differences of caecal microbiota between lines through an action on immunity. At least one genetics study showed that several quantitative trait loci (QTL) controlling higher abundance of specific bacterial species might be involved in immunity^48^. The present lines differ for their MHC, which has a pivotal role in the adaptive immune response. In fact, MHC may shape the intestinal microbiota through its influence on the antibody response, on some CD4+ Th cell subsets, or on innate lymphoid cells that recognize commensal antigens^48^. Its involvement in the gut enterocytes, that secrete antimicrobial molecules able to influence microbiota composition, is also suspected^48–50^. Furthermore, differences in both affinity and number of amino acids recognized in binding pockets between alleles of MHC class I molecules in birds is a new concept that could explain the association between MHC haplotypes and some disease resistances^51^. According to this concept, MHC motifs can be considered as either “promiscuous” or “fastidious”. Alleles leading to a lower expression of MHC class I molecules having a lower affinity but a greater ability to bind a large variety of peptides are identified as promiscuous (generalist) against a wide variety of infectious pathogens. At the opposite, fastidious alleles display a higher expression of MHC class I molecules that can bind to a reduced variety of peptides, which can support an advantage against some specific pathogens. In a study on wild seabirds, but with limited sampling, an effect of the MHC on the gut microbiota was observed^52^. In stickleback fish, 6 out of 14 MHC motifs from class II molecules were significantly associated with the abundance of 7 bacterial families, and fishes with more diverse MHC motifs had less diverse gut microbiota^49^. Even if it is difficult to compare effects of the MHC on the gut microbiota between poultry and fish, which harbor quite dissimilar bacterial communities and environments, we can notice that the *Enterobacteriaceae* family was prone to sex-specific MHC effects in stickleback fish. Interestingly, in a similar way, we also observed in the present study that OTU from this family displayed higher relative abundance in the B14 and B12 lines. Furthermore, *Enterobacteriaceae* is a group of interest as many bacteria from this family are pathogenic and lead to intestinal inflammation^53^. In mice, 189 OTU displayed differential abundances between animals differing genetically at the MHC locus^50^. In poultry, and more specifically in layers, only one similar study was performed on the caecal microbiota from two congenic inbred lines that differ by their MHC genotypes^54^. Alpha-diversity was the only metric reported to describe the MHC effect, and as we observed in the present study, they did not notice any significant difference in Shannon’s diversity in the microbiota composition of the two lines. To our knowledge, there is no other publication about the association between MHC haplotypes and gut microbiota in poultry and the present study thus brings some promising elements to deeply investigate the effect and role of MHC in outbred populations, segregating for different MHC haplotypes.

### A set of correlations between immune phenotypes and caecal microbiota composition

Larsen et al. reported a large variation in blast transformation between MHC-B lines for γδ T cells considering the same bird population^29^. In the present study, TCR_ϒδ_ expression on TCR_γδ_ T cells was also the immune parameter with the greatest consistency among the different association analyses we performed between the microbiota and the immune phenotypes. It was particularly associated with the caecal microbiota of the B21, B14 and B15 lines that are of interest to further study potential interactions. TCR_ϒδ_ expression on TCR_γδ_ T cells also shared negative associations with many OTU from the *Escherichia*-*Shigella* genus, whatever the level of infection in both the control and the vaccinated group, and especially with Cluster 544 from this same genus. Such consistency strengthens the likelihood of a biological link between a specific bacteria from the *Escherichia-Shigella* genus and TCR_ϒδ_ expression on TCR_γδ_ T cells. Gamma delta T cells are a subpopulation of T lymphocytes with TCR composed by ϒ and δ chains. In adult chickens, ϒδ T cells are the dominant population among intestinal epithelial lymphocytes (20 to 60% of the lymphocytes)^55^, circulating T cells (20 to 50%), and splenocytes (30%)^56^. They are also contributing to the first line of defense against pathogens^55^. This population grows during infectious challenges with *Salmonella enterica*^57^, and demonstrates in vitro a capacity for cytokine and interferon production (IFNϒ, TNFα, IL17). Their dependency on APC and MHC restriction for antigen recognition remains uncertain, and some ϒδ T cells recognizing directly bacterial and viral epitopes were identified^55^. In mice, intestinal *lamina propria*, γδ T cells are the major source of IL-17, which is gut protective as it promotes the repair of damaged intestinal epithelium through the regulation of occludin subcellular localization to cell junctions^58,59^. Furthermore, some reports in mouse show that lipid antigens from *E. coli* are recognized by the hepatocytes CD1d, which alleviates hepatic γδ T-17 cell clones^60^. In the present study, we observed that several OTU from *Eschericha-Shigella* genus were negatively correlated with TCR_ϒδ_ expression on TCR_γδ_ T cells, and were also less abundant in the B2 and B21 lines that were characterized as high responders to the IBV vaccination. Despite the absence of known orthologous gene of CD1d in the chicken genome, we can wonder if the CD1 paralogous genes could be involved in a similar mechanism^61^. Thus, in the present study, we can hypothesize that bacteria from the *Escherichia-Shigella* genus increased the systemic level of bacterial lipid antigens, which subsequently mitigated poultry γδ T cells.

## Conclusion

In the present study, performed on inbred chicken lines with contrasted abilities to respond to IBV vaccination, we observed after an IBV infection that the different genetic lines harbored dissimilar microbiota. In addition to this role of the host genetics on the microbiota composition, we observed that the microbiota from the vaccinated chickens was highly dissimilar from the microbiota of the non-vaccinated ones. We hypothesized that a bi-directional crosstalk may have occurred between immunity and the gut microbiota. Vaccinated animals, that had developed good protective immunity, displayed a different immune response upon challenge, which may have dampened the shift of their gut microbiota during the infection. On the contrary, the non-vaccinated animals, which encountered IBV for the first time, encountered a higher immune perturbation, which in conjunction with dyspnea, polydipsia, and a reduced appetite may have fostered the shift of their gut microbiota. Regardless of the vaccine status and the ability to respond to IBV vaccination, we observed that some bacteria from the *Escherichia*-*Shigella* genus were always negatively correlated with TCR_ϒδ_ expression on circulating TCR_γδ_ T cells and were less abundant in the B2 and B21 lines, that displayed the best vaccine efficiency. Those bacteria may affect the γδ T cell differentiation or proliferation, and thus their subsequent function e.g. cytokine production.

## Methods

### Animals

Offspring from 6 different inbred lines kept at Aarhus University and homozygous for the MHC haplotypes B2, B12, B14, B15, B19, and B21 were used for this experiment, as described previously^26^. Briefly, a group of 48 vaccinated chickens, with 8 chickens per line, was reared in two pens (24 chickens per pen, 4 chickens of each line per pen) in a unit of a bio-secure IBV-free facility. Vaccination was performed nasally at 2 week of age with a live attenuated IBV vaccine (Nobilis IB Ma5, MSD Animal Health) with a booster vaccination at 5 weeks of age. Another group of chickens with the same composition (n = 48; 8 chickens per line) was reared in another unit from the same facility, with separate entrances and strict biosafety precautions, but not vaccinated. All the vaccinated and non-vaccinated chickens were nasally challenged at 8 weeks of age with the IBV strain M41, as described previously^29^, generating two groups: vaccinated and challenged (V-Ch), and non-vaccinated and challenged (NV-Ch) (Fig. 1). Within the present population of 96 animals, all but 6 were females. The 6 males were distributed in different groups: 3 males from the B21 line (2 NV-Ch and 1 V-Ch) and 3 males from the B2 line (1 NV-Ch and 2 NV-Ch). Progenitors of the studied animals were reared in the same environment. Chickens were fed ad libitum with a wheat, soy, oat, corn, and barley diet (Paco Start 19, Forrådshuset, dlg, Denmark) adapted to their nutritional requirements as defined by the National Research Council requirements (NRC, 1994) (18.5% of crude proteins; 3.3% of crude lipids, 4.7% of crude fiber).

The experiment was conducted under protocols approved by the Danish Animal Experiments Inspectorate and complied with the Danish Ministry of Justice Law number 382 (10th June 1987) and Acts 739 (6thDecember 1988) and 333 (19th May 1990) concerning animal experimentation and care of experimental animals, following the described ethical guidelines. Ethics committee of Aarhus University approved the study, and license to conduct the animal experiment was obtained by DVM Ricarda Engberg, Aarhus University (license number 2017-15-0201-01211). The study was carried out in compliance with ARRIVE guidelines.

### Samples collection

Tracheal swabs were collected at 0, 3, 4, and 5 days post IBV infection to assess IBV viral load. At 9 weeks of age, and 7 days post infection, the 96 animals were slaughtered by cervical dislocation in conformity with the Danish regulation for experimental animals. Animals were weighted before slaughter. After slaughter, caecal contents were gently removed from the caecal bags to avoid collection of mucosa and immediately frozen in liquid nitrogen before being stored at − 80 °C. EDTA-preserved blood samples were collected at the same time from the jugular vein for the immuno-phenotyping of all the chickens.

### Immunoprofiling, serum titers and viral load

As reported previously^29^, serum IBV antibody titers were assessed by ELISA and peripheral blood leukocyte counts by flow cytometry one week post IBV infection. The following cell subsets were included in the analyses: heterophils (CD45+ SSChigh), monocytes (CD45+ Kul01+), thrombocytes (CD45+ CD41/61+), B cells (CD45 + Bu1 + MHCII + IgM +) and various T cell subsets (TCRγδ-CD4 + CD25 ± , TCRγδ-CD8β + CD25 ± , TCRγδ + CD8β + /− CD25 ±).

The tracheal IBV viral load was measured from tracheal swabs collected at 0, 3, 4, and 5 days post IBV infection, by RT-qPCR.

### DNA extraction, amplification and sequencing

DNA was extracted from individual caecal contents, using a standardized protocol adapted from Godon et al^62^. DNA quantities were measured using a Qubit analyser, and DNA qualities using a Nanodrop analyser by measuring ratios of absorbances at 230, 260 and 280 nm. DNA samples were diluted at 15 ng/µL before PCR amplification. A 464 bp fragment targeting the hypervariable V3–V4 region from bacterial 16S rRNA gene was first amplified with the following primers: 460-F-iII-16S (adapter-CTTTCCCTACACGACGCTCTTCCGATCT-343-ACGGRAGGCAGCAG-357), and 460-R-iII-16S (adapter-GGAGTTCAGACGTGTGCTCTTCCGATCT-781-TACCAGGGTATCTAATCCT-806), in accordance with primer adapter previously reported by Lluch et al^63^. The first PCR reaction was conducted with 1 µL of genomic DNA and 0.5 µL of each primer (10 µM), 0.5 µL of dNTP mix (10 mM), 2.5 µL of 10X MTP Taq buffer mix (10 mM), 0.25 µL of MTP Taq DNA Polymerase (SIGMA-ALDRICH) and water qsp 25 µL. PCR conditions were: initial denaturation at 94 °C for 1 min, followed by 30 cycles of 94 °C for 1 min, annealing at 65 °C for 1 min, extension at 72 °C for 1 min, then a final elongation step at 72 °C for 10 min. We checked by agarose gel electrophoreses that each PCR generated a unique product size at the expected length. Purifications, second PCR with the Illumina adapters, and sequencing using a 2 × 300 bp MiSeq Illumina sequencer were performed by the GeT-PlaGe platform (INRAE, Toulouse). DNA from three samples could not be extracted, leading to a set of 93 animals with exploitable microbiota DNA. Raw sequences are available through the accession PRJNA658073 on the NCBI SRA (Sequence Read Archive).

### Bioinformatic analyses

We used the FROGS pipeline (v3.0)^64^ to assemble read pairs, trim the 460-F-iII-16S and 460-R-iII-16S primers, and filter sequences to keep only amplicons with a length ranging from 300 to 490 bp. Unique sequences with their associated abundances in each sample were clustered with FROGS using Swarm (v 1.4.1)^65^, first with an aggregation distance of 1 and secondly of 3. FROGS removes chimera thanks to Vsearch (v2.6.0)^66^. Rare OTU were filtered out (abundance < 0.005% of total abundance)^67^ and the remaining sequences were annotated by NCBI Blast + (2.6.0)^68^ with an alignment on SILVA database restricted to 16S references (version 132 from 2017 update)^69^. We obtained an abundance table and then produced a phylogenetic tree using FROGS Tree tool with Mafft (v7.310)^70^ and Fasttree (2.1.10)^71^. We rarefied counts for α- and β-diversity analyses at 10,000 counts per sample. The unrarefied OTU table, the corresponding taxonomic classifications, and the metadata are available in the Supplementary Tables S13, S14, and S15 respectively.

### Bio-statistical analyses

#### Microbiota taxonomic composition and diversity

Bio-statistical analyses were performed using R 3.5.2 and the Phyloseq 1.26.1 package. From the distribution of OTU counts, we described taxonomic composition at phylum, family and genus levels. To identify excluding or co-abundant genera, we calculated Spearman correlations between the abundances of each possible pair of genera on the overall dataset with the Hmisc 4.2.0 package. We represented on a network the moderate and high associations present in at least 20% of the sampled chicken, using the cutoff of 0.5 for the Spearman coefficient, and 0.05 for the adjusted p-value.

#### Effect of vaccination and chicken line on microbiota composition

We used the Vegan 2.5.5 package to evaluate the α-diversity by calculating the species richness, which is the number of observed OTU, and the Chao1, Shannon, and Inverse Simpson indexes that we represented for each vaccine group and each line. β-diversity indices were calculated using the Bray–Curtis (BC) dissimilarities. We described β-diversity in each line and represented it using a NMDS analysis (Non metrical Multidimensional Scaling). We first depicted the effect of vaccination by gathering the 6 chicken lines, and then in each line separately. We represented the vaccination impact in the different lines, adding on the NMDS ordination the centroids displacements from the control group to the vaccinated one in each line. To assess the effects of line and vaccination on α-diversity, we performed an ANOVA on α-indices. We used the vaccine status as a co-variable to assess the line effect, and the line as a co-variable to assess the vaccine-status effect. We did not correct the data for a “sex” effect, because : only 6 out of 93 animals were males, they were distributed in different groups, and we assessed with an NMDS analysis that the microbial communities of males and females were not dissimilar (Adonis R^2^ = 0.008, p = 0.54). Similarly, we did not correct the data for a putative pen effect, because in this experiment it was confounded with the vaccine status group. Significance threshold was set at 0.05 for the p-values. To assess the line and the vaccination effects on β-diversity, we used an Adonis model in Vegan. Similarly, we used the MetagenomeSeq 1.24.1 package to compare OTU abundances between each couple of lines, and between each couple of vaccine status groups. This package enables the normalization of raw sequences counts through a cumulative-sum scaling (CSS) method to reduce biases due to uneven sequencing depth^72^. We then used a zero-inflated Gaussian mixture model with vaccine status or line as main effect to identify, respectively, OTU with an abundance differing between each pair of lines, or rather between each vaccine status, with a threshold of 0.05 on the adjusted p-value (FDR). Analyses were performed independently at the species and genus levels.

#### Association between microbiota and immune phenotypes

We searched for associations between individual caecal microbiotas (OTU) and the immune phenotypes by calculating Spearman correlations between each immune parameter and the abundance of each OTU. To perform it, we used as starting point the normalized abundances from previous MetagenomeSeq normalization. As both the vaccination and the line had a significant effect on the microbiota, we collected residuals from linear models implemented on those normalized OTU counts with lines and vaccine status as main effects. Similarly, we collected residuals from linear models implemented on each immune parameter with the vaccine status and line as main effects. Then, these two sets of residuals were used to calculate Spearman correlation coefficients, and we set a cutoff of 0.05 on their adjusted p-values.

We first performed this analysis on the whole dataset, and then on the vaccinated challenged (V-Ch) and on the non vaccinated challenged groups (NV-Ch) separately. To analyse the V-Ch and NV-Ch groups separately, we collected residuals from linear models implemented on each normalized OTU count and each immune parameter with only the line as main effect. We thus assessed the vaccine effect from the comparison of the separate results. We represented the intermediate and high associations on a network, adjusting the cutoff on the Spearman correlation in order to keep at least 5 significant associations. Thus, we set a cutoff of 0.4 on the entire dataset, and a cutoff raised to 0.5 on the analysis performed in the V-Ch and the NV-Ch groups separately.

We also searched for associations between the microbiota and the immune phenotypes regarding the effect of microbiota β-diversity calculated with Bray–Curtis distances on each immune parameter. We used an Adonis model on the entire dataset with line and vaccine status as main effects. In order to identify the lines that contributed the most to the observed effects, we performed these analyses on each line separately by implementing the Adonis model only with the vaccine status as main effect. For the immune parameters that displayed the highest association with the microbiota, we represented their level according to the microbiota ordination on a NMDS plot.

## Supplementary Information

Supplementary Information.

Supplementary Tables.

## Data Availability

Raw sequences are available through the accession PRJNA658073 on the NCBI SRA (Sequence Read Archive), and the sequences of negative control were included as Additional File 1. The unrarefied OTU table, the corresponding taxonomic classifications and the metadata table have been included as Additional Files 2, 3, and 4 respectively.

## Abbreviations

AGP: Antibiotics as growth promoters
APC: Antigen-presenting cells
BC: Bray–Curtis
CAP: Cationic antimicrobial peptide
FDR: False discovery rate
IAV: Influenza A virus
IBV: Infectious bronchitis virus
ILCs: Intestinal innate lymphoid cells
IFN: Interferon
LPS: Lipopolysaccharide
MHC: Major histocompatibility complex
NMDS: Non metrical multidimensional scaling
NV-Ch: Non vaccinated challenged
PRRs: Pattern recognition receptors
QTL: Quantitative trait loci
RSV: Respiratory syncytial virus
TLR: Toll like receptors
V-Ch: Vaccinated challenged
