# Supplementary material for: Caecal microbiota composition of experimental inbred MHC-B lines infected with IBV differs according to genetics and vaccination
Source: Sci Rep. 2022 Jun 15;12:9995. doi: 10.1038/s41598-022-13512-7 (PMC9199466; doi:10.1038/s41598-022-13512-7)
Supplement: Supplementary file 1 — Supplementary Information. [file 41598_2022_13512_MOESM1_ESM.docx]

**Supplementary Information**


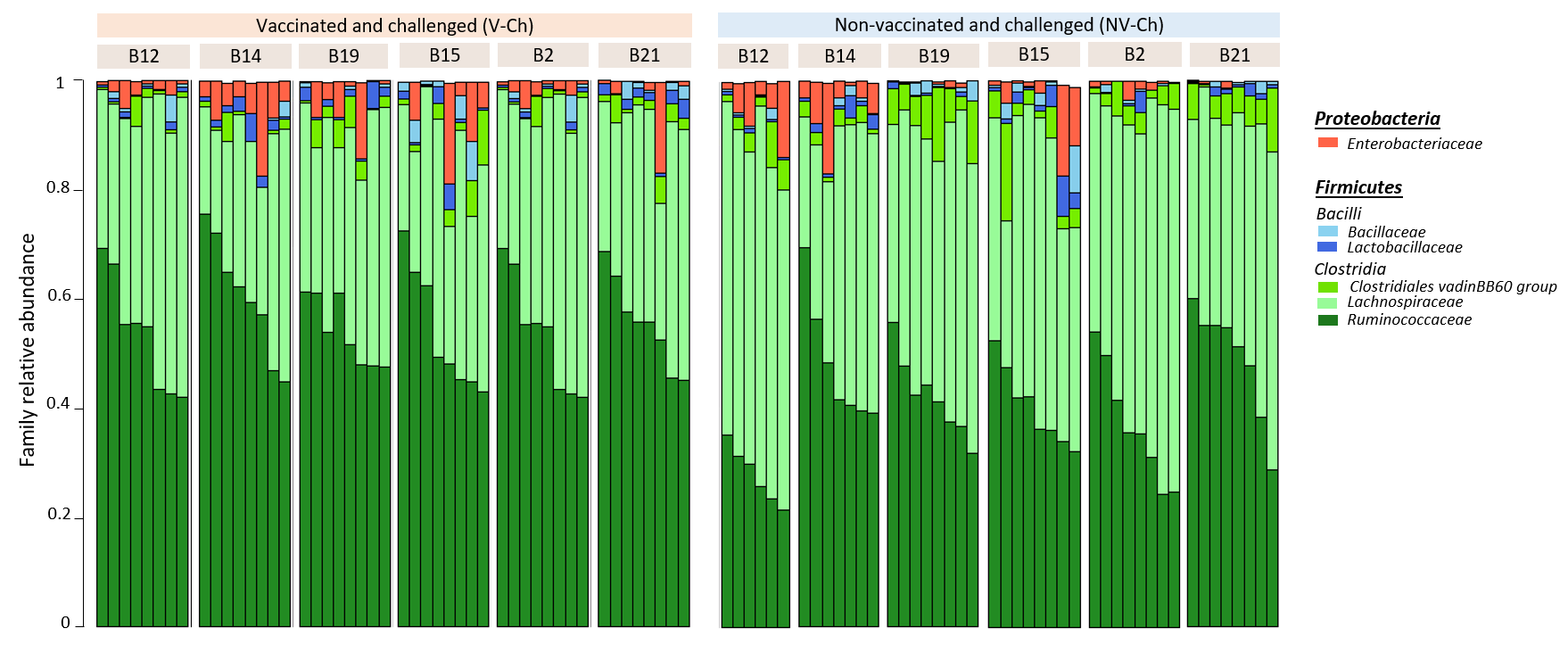


**Supplementary Fig. S1**: **Caecal microbial community of 9-week-old layers, one week after their infection with IBV**. Taxonomic composition is given at the family level among the different lines, and according to their vaccine status: vaccinated and challenged (V-Ch) vs non-vaccinated and challenged (NV-Ch) (n=93). Only taxons with a mean relative abundance above 2.0% are displayed.


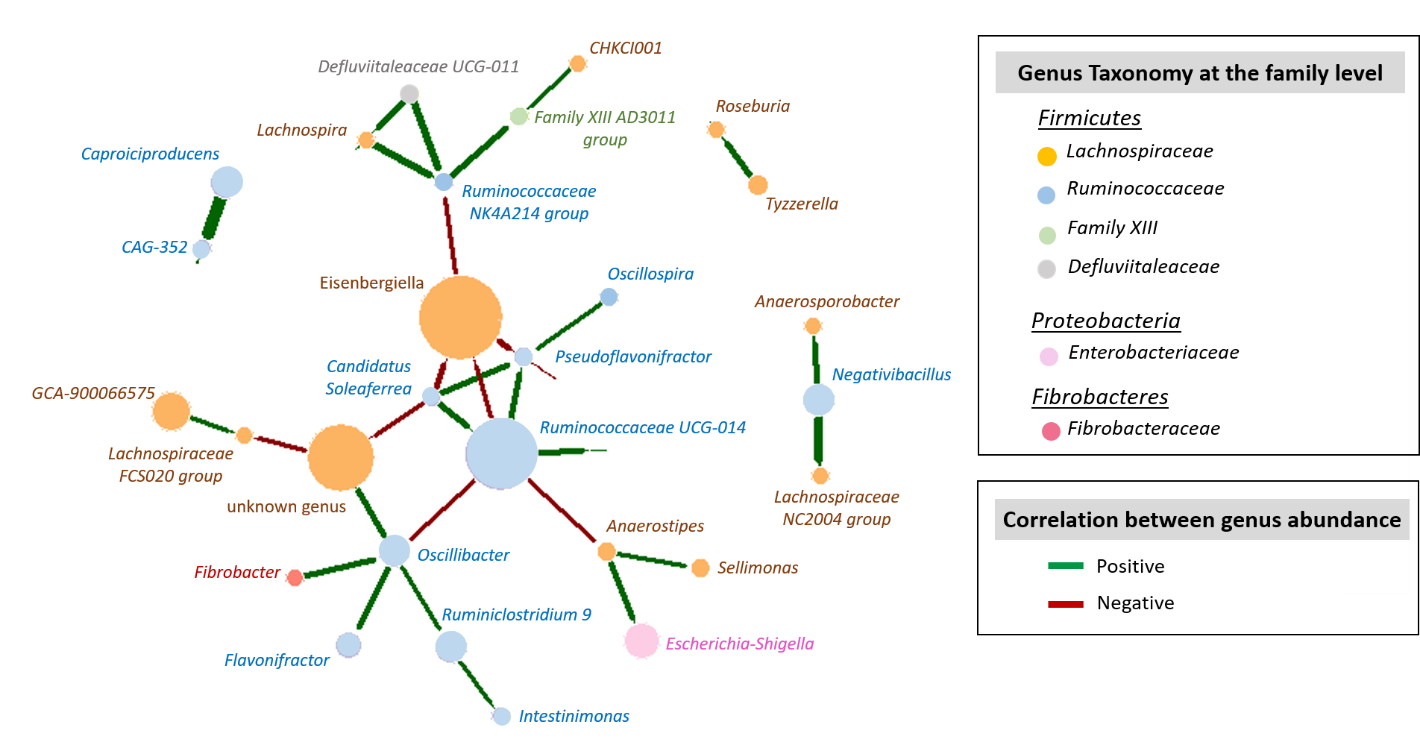


**Supplementary Fig. S2:** **Co-abundance network in the caecal microbiota of the chickens, one week after their infection with IBV**. Co-abundances were calculated through spearman correlation coefficient on the genus relative abundances in the total population (n=93). Only intermediate (Spearman correlation coefficient > 0.4) or high (Spearman correlation coefficient > 0.5) correlations with statistical significance (p-value<0.05) are represented. Each genus is represented by a node with a color that corresponds to the family affiliation. The co-abundances between two genera are represented by connecting lines, either green or red for positive and negative correlations, respectively. All correlation calculations and their associated p-values are available in **supplementary Table S2**.


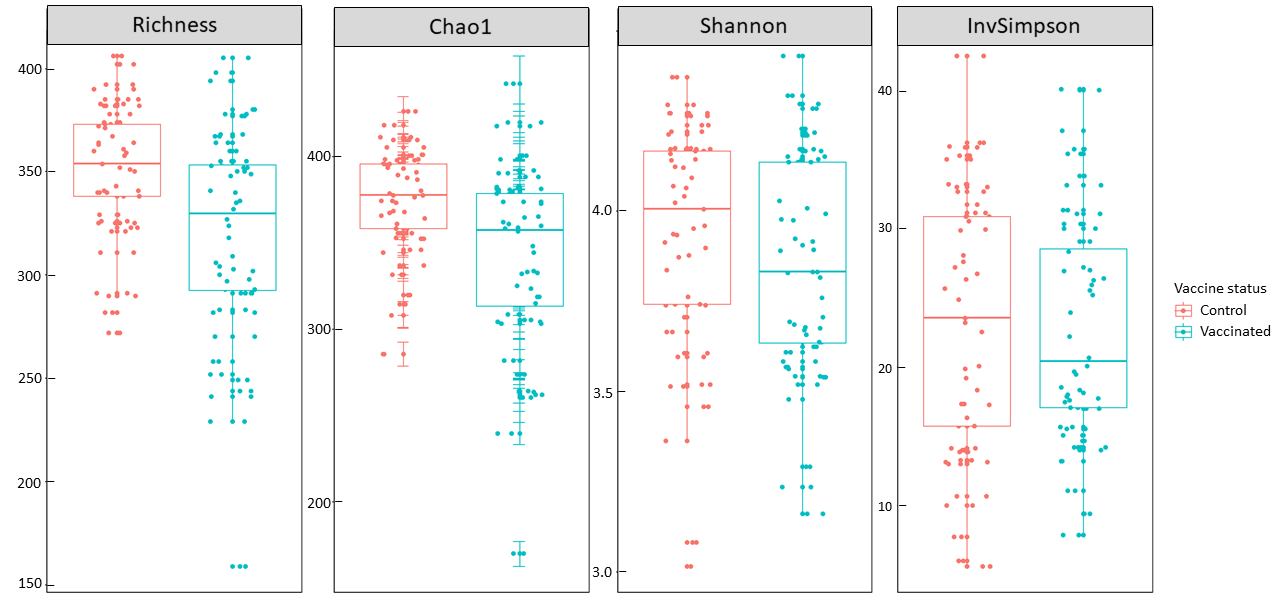


**Supplementary Fig. S3**: **Line effect on α-diversity of 9-week-old layers, one week after their infection with IBV**. Boxplots of Richness, Chao1, Shannon and InvSimpson indices among the lines in the vaccinated (V-Ch) and non-vaccinated (NV-Ch) groups considered together. Alpha diversity indices are available in **Supplementary Table S5**.


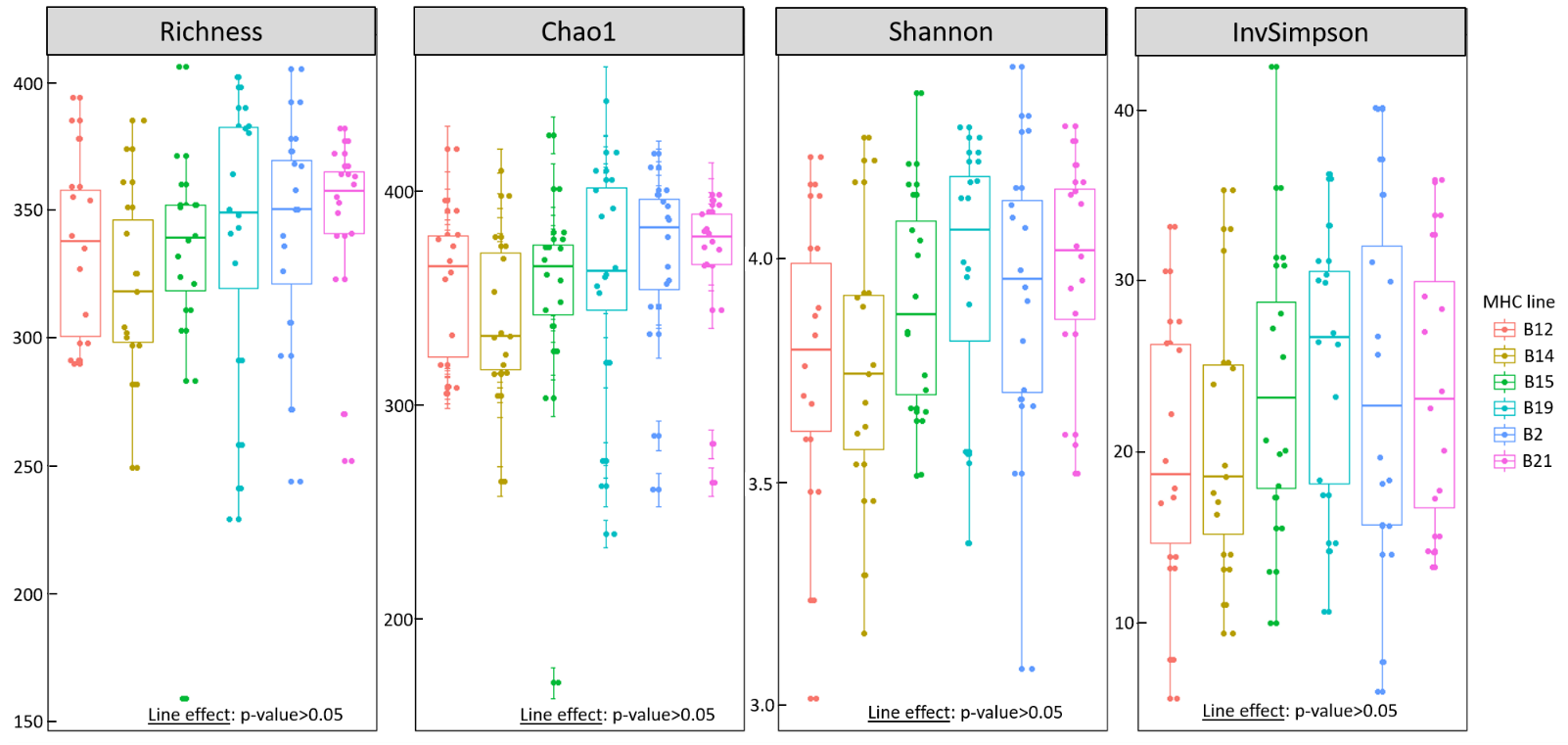


**Supplementary Fig. S4**: **Vaccination effect on α-diversity of 9-week-old layers, one week after their infection with IBV**. Boxplots of Richness, Chao1, Shannon and InvSimpson indices among in the vaccinated and challenged (V-Ch, pink dot) and non-vaccinated and challenged (NV-Ch, blue dot) groups. Alpha diversity indices are available in **Supplementary Table S5**.


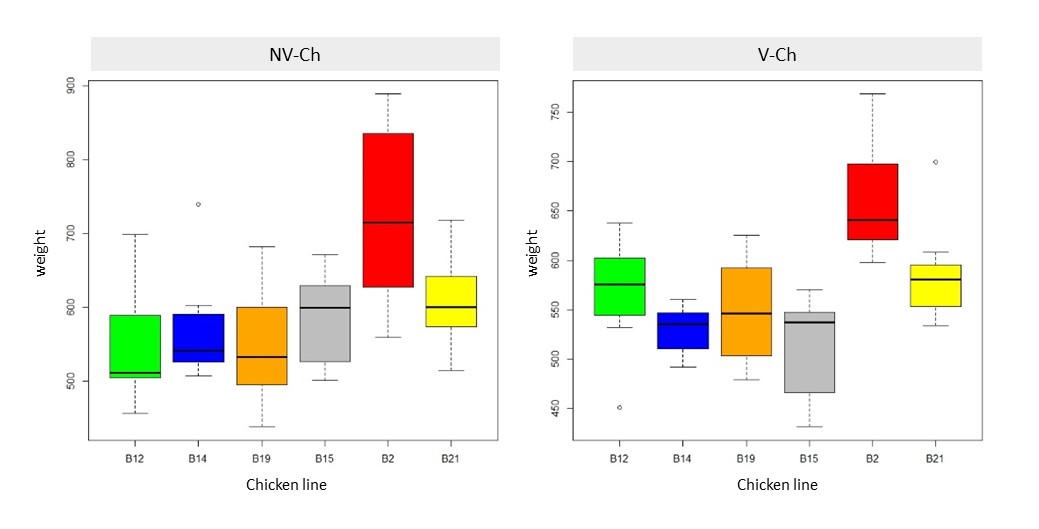


**Supplementary Fig. S5**: **Weight of 9-week-old layers, one week after their infection with IBV**. Weight (g) is given the day of slaughter among the different lines, and according to their vaccine status: vaccinated and challenged (V-Ch) vs non-vaccinated and challenged (NV-Ch) (n=93).


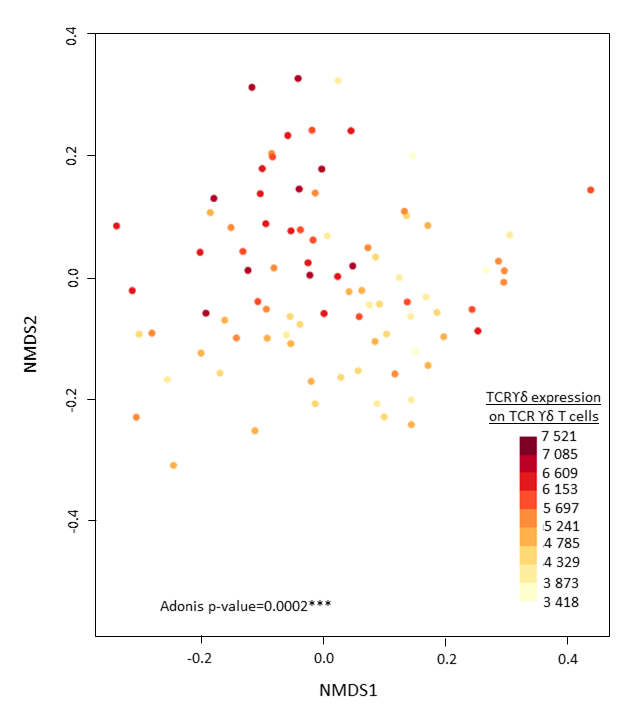


**Supplementary Fig. S6:** **Association between chicken caecal microbiota and TCR_ϒδ_ on TCR_ϒδ_^+^ T cells in laying hens, one week after their infection with IBV**. NMDS representation of the caecal microbiota with their individual levels of TCR_ϒδ_ on TCR_ϒδ_^+^ T cells (points with a gradient of color representing the level of TCR_ϒδ_ expression on TCR_δϒ_ T cells).

**Supplementary Table S2:** Co-abundance network at the genus level. Correlation and their associated p-value between relative abundances of each couple of genus from the cecal microbiota of laying hens that displayed a significant correlation. Unknown annotation at the genus level are given at a lower rank: (f) family, or (c) class.

| **Correlation** | **Couple of genera** | | **Correlation** | **p-value** | **FDR** |
| --- | --- | --- | --- | --- | --- |
| Positvie | *Escherichia-Shigella* | Gammaproteobactria (c) | 0.95 | 0.00E+00 | 0.00E+00 |
|  | *CAG-352* | Ruminococcaceae (f) | 0.87 | 0.00E+00 | 0.00E+00 |
|  | *CAG-352* | *Caproiciproducens* | 0.80 | 0.00E+00 | 0.00E+00 |
|  | *Caproiciproducens* | Ruminococcaceae (f) | 0.77 | 0.00E+00 | 0.00E+00 |
|  | *Anaerosporobacter* | *Negativibacillus* | 0.72 | 4.44E-16 | 2.27E-13 |
|  | *Ruminococcaceae NK4A214 group* | *Defluviitaleaceae UCG-011* | 0.66 | 5.60E-13 | 2.38E-10 |
|  | *Ruminococcaceae NK4A214 group* | *Lachnospira* | 0.64 | 4.28E-12 | 1.56E-09 |
|  | *Ruminococcaceae UCG-014* | *Candidatus Soleaferrea* | 0.61 | 1.29E-10 | 4.13E-08 |
|  | *Lachnospira* | *Defluviitaleaceae UCG-011* | 0.59 | 4.65E-10 | 1.32E-07 |
|  | *Pseudoflavonifractor* | *Ruminococcaceae UCG-014* | 0.59 | 5.65E-10 | 1.44E-07 |
|  | *Lachnospiraceae NC2004 group* | *Negativibacillus* | 0.58 | 8.32E-10 | 1.93E-07 |
|  | *Oscillibacter* | *Fibrobacter* | 0.58 | 1.21E-09 | 2.59E-07 |
|  | *Pseudoflavonifractor* | *Candidatus Soleaferrea* | 0.58 | 1.53E-09 | 3.02E-07 |
|  | *Oscillibacter* | unknown genus | 0.57 | 1.76E-09 | 3.22E-07 |
|  | Bacillaceae (f) | *Ruminococcaceae UCG-014* | 0.57 | 2.25E-09 | 3.83E-07 |
|  | Gammaproteobactria (c) | *Anaerostipes* | 0.56 | 4.95E-09 | 7.90E-07 |
|  | *Flavonifractor* | *Oscillibacter* | 0.55 | 8.96E-09 | 1.20E-06 |
|  | *Family XIII AD3011 group* | *Ruminococcaceae NK4A214 group* | 0.55 | 1.12E-08 | 1.43E-06 |
|  | *Roseburia* | *Tyzzerella* | 0.55 | 1.25E-08 | 1.53E-06 |
|  | *Ruminiclostridium 9* | *Oscillibacter* | 0.54 | 1.93E-08 | 2.24E-06 |
|  | *Escherichia-Shigella* | *Anaerostipes* | 0.54 | 2.48E-08 | 2.76E-06 |
|  | *Intestinimonas* | *Ruminiclostridium 9* | 0.54 | 2.93E-08 | 3.12E-06 |
|  | *Sellimonas* | *Anaerostipes* | 0.53 | 4.10E-08 | 4.19E-06 |
|  | *Lachnospiraceae FCS020 group* | *GCA-900066575* | 0.52 | 6.94E-08 | 6.82E-06 |
|  | *Pseudoflavonifractor* | *Oscillospira* | 0.52 | 1.01E-07 | 8.59E-06 |
|  | *Family XIII AD3011 group* | *CHKCI001* | 0.51 | 2.36E-07 | 1.82E-05 |
| Negative | *Eisenbergiella* | *Candidatus Soleaferrea* | -0.56 | 5.76E-09 | 8.66E-07 |
|  | *Pseudoflavonifractor* | *Eisenbergiella* | -0.56 | 6.64E-09 | 9.42E-07 |
|  | unknown genus | *Candidatus Soleaferrea* | -0.52 | 7.87E-08 | 7.35E-06 |
|  | *Ruminococcaceae NK4A214 group* | *Eisenbergiella* | -0.52 | 8.05E-08 | 7.35E-06 |
|  | *Eisenbergiella* | *Ruminococcaceae UCG-014* | -0.52 | 8.48E-08 | 7.47E-06 |
|  | *Anaerostipes* | *Ruminococcaceae UCG-014* | -0.52 | 1.19E-07 | 9.78E-06 |
|  | *Lachnospiraceae FCS020 group* | unknown genus | -0.51 | 1.95E-07 | 1.55E-05 |
|  | *Oscillibacter* | *Ruminococcaceae UCG-014* | -0.50 | 3.18E-07 | 2.39E-05 |

**Supplementary Table S4:** Effect of IBV vaccination on OTU relative abundance in caecal microbiota of each MHC line.

The p-value, and adjusted p-value of the difference is given, and we indicated the mean relative abundance of each OTU in the vaccinated and not vaccinated (control) group. OTU taxonomy is given at the phylum, family, and genus level when available, with '-' indicating that the annotation is the same than the taxonomy of the OTU above.

| **Line** | **OTU** | **phylum** | **class** | **family** | **genus** | **p-values** | **FDR** | **Mean relative abundance** | |
| --- | --- | --- | --- | --- | --- | --- | --- | --- | --- |
| **(OTU number)** |  |  |  |  |  |  |  | **V-Ch** | **NV-Ch** |
| **B2-line (39 OTUs)** | Cluster_25 | *Firmicutes* | *Clostridia* | *Lachnospiraceae* | *Eisenbergiella* | 2.19E-05 | 2.80E-03 | 6.01E-02 | 2.68E-03 |
|  | Cluster_157 | *-* | *-* | *-* | *-* | 7.92E-04 | 1.61E-02 | 2.42E-03 | 1.00E-04 |
|  | Cluster_480 | *-* | *-* | *-* | *Lachnospiraceae NK4A136 group* | 8.93E-04 | 1.63E-02 | 6.08E-04 | 3.21E-05 |
|  | Cluster_683 | *-* | *-* | *-* | *-* | 4.13E-03 | 4.29E-02 | 3.80E-04 | 2.78E-05 |
|  | Cluster_96 | *-* | *-* | *-* | *Ruminococcus torques group* | 2.71E-03 | 3.61E-02 | 9.82E-04 | 6.93E-04 |
|  | Cluster_57 | *-* | *-* | *-* | *-* | 2.45E-03 | 3.61E-02 | 8.62E-03 | 2.43E-03 |
|  | Cluster_190 | *-* | *-* | *-* | unknown genus | 2.77E-04 | 1.03E-02 | 3.06E-03 | 2.09E-04 |
|  | Cluster_151 | *-* | *-* | *-* | *Tyzzerella 3* | 2.54E-03 | 3.61E-02 | 8.21E-04 | 2.03E-04 |
|  | Cluster_219 | *-* | *-* | *-* | *Fusicatenibacter* | 3.36E-03 | 4.08E-02 | 1.17E-03 | 2.04E-04 |
|  | Cluster_722 | *-* | *-* | *-* | *Anaerosporobacter* | 4.57E-03 | 4.50E-02 | 2.32E-05 | 1.90E-04 |
|  | Cluster_481 | *-* | *-* | *-* | *Lachnoclostridium* | 2.93E-03 | 3.76E-02 | 4.52E-04 | 4.06E-05 |
|  | Cluster_77 | *-* | *-* | *-* | unknown genus | 7.97E-04 | 1.61E-02 | 1.06E-02 | 8.89E-04 |
|  | Cluster_94 | *-* | *-* | *-* | unknown genus | 2.76E-04 | 1.03E-02 | 6.03E-03 | 8.62E-04 |
|  | Cluster_559 | *-* | *-* | *-* | unknown genus | 2.72E-03 | 3.61E-02 | 2.77E-04 | 4.77E-05 |
|  | Cluster_53 | *-* | *-* | *-* | unknown genus | 3.40E-03 | 4.08E-02 | 5.10E-03 | 2.95E-03 |
|  | Cluster_317 | *-* | *-* | *-* | *Lachnospiraceae UCG-008* | 1.83E-04 | 1.00E-02 | 5.75E-04 | 4.09E-05 |
|  | Cluster_106 | *-* | *-* | *-* | *-* | 4.09E-03 | 4.29E-02 | 3.26E-03 | 1.72E-03 |
|  | Cluster_247 | *-* | *-* | *-* | *-* | 2.72E-04 | 1.03E-02 | 1.08E-04 | 1.47E-03 |
|  | Cluster_42 | *-* | *-* | *-* | *Lachnospiraceae FE2018 group* | 3.82E-04 | 1.05E-02 | 2.31E-03 | 1.20E-02 |
|  | Cluster_205 | *-* | *-* | *-* | *Blautia* | 8.44E-04 | 1.62E-02 | 1.78E-05 | 5.96E-04 |
|  | Cluster_112 | *-* | *-* | *-* | unknown genus | 4.91E-04 | 1.26E-02 | 3.49E-05 | 3.13E-03 |
|  | Cluster_198 | *-* | *-* | unknown family | unknown genus | 2.10E-03 | 3.37E-02 | 1.46E-04 | 5.05E-04 |
|  | Cluster_6 | *-* | *-* | *Ruminococcaceae* | *Faecalibacterium* | 3.34E-04 | 1.03E-02 | 8.45E-05 | 2.55E-01 |
|  | Cluster_39 | *-* | *-* | *-* | *Ruminococcaceae UCG-014* | 2.15E-08 | 8.26E-06 | 4.40E-04 | 2.88E-02 |
|  | Cluster_228 | *-* | *-* | *-* | *-* | 9.05E-05 | 5.96E-03 | 3.05E-05 | 1.43E-03 |
|  | Cluster_152 | *-* | *-* | *-* | *-* | 1.27E-03 | 2.11E-02 | 1.05E-04 | 3.82E-03 |
|  | Cluster_125 | *-* | *-* | *-* | *-* | 7.56E-04 | 1.61E-02 | 2.45E-04 | 5.23E-03 |
|  | Cluster_489 | *-* | *-* | *-* | *-* | 3.88E-03 | 4.29E-02 | 4.68E-05 | 6.49E-04 |
|  | Cluster_164 | *-* | *-* | *-* | *-* | 2.38E-03 | 3.61E-02 | 2.14E-03 | 9.14E-05 |
|  | Cluster_313 | *-* | *-* | *-* | *Oscillibacter* | 6.87E-05 | 5.96E-03 | 1.35E-03 | 1.29E-04 |
|  | Cluster_204 | *-* | *-* | *-* | *-* | 3.67E-03 | 4.27E-02 | 1.23E-03 | 1.76E-04 |
|  | Cluster_174 | *-* | *-* | *-* | *Ruminiclostridium 9* | 9.24E-06 | 1.77E-03 | 1.63E-03 | 1.21E-04 |
|  | Cluster_110 | *-* | *-* | *-* | *Ruminiclostridium 5* | 4.41E-03 | 4.46E-02 | 2.49E-03 | 9.95E-04 |
|  | Cluster_143 | *-* | *-* | *-* | *Flavonifractor* | 3.49E-04 | 1.03E-02 | 1.18E-03 | 5.41E-05 |
|  | Cluster_116 | *-* | *-* | *-* | unknown genus | 3.01E-04 | 1.03E-02 | 2.36E-03 | 4.85E-04 |
|  | Cluster_154 | *-* | *-* | *-* | unknown genus | 5.53E-04 | 1.33E-02 | 1.19E-04 | 1.13E-03 |
|  | Cluster_137 | *-* | *-* | *-* | unknown genus | 4.08E-03 | 4.29E-02 | 2.32E-04 | 3.17E-03 |
|  | Cluster_121 | *-* | *-* | *Clostridiales vadinBB60 group* | unknown genus | 1.25E-03 | 2.11E-02 | 1.23E-04 | 3.59E-03 |
|  | Cluster_54 | *-* | *Bacilli* | *Lactobacillaceae* | *Lactobacillus* | 9.31E-05 | 5.96E-03 | 4.41E-03 | 7.75E-04 |
| **B12-line (4 OTUs)** | Cluster_84 | *Firmicutes* | *Clostridia* | *Lachnospiraceae* | unknown genus | 1.62E-04 | 0.02715939 | 0.001180499 | 0.000112162 |
|  | Cluster_6 | *-* | *-* | *Ruminococcaceae* | *Faecalibacterium* | 3.72E-07 | 0.00012466 | 0.000125022 | 0.364558053 |
|  | Cluster_21 | *-* | *-* | *-* | *Ruminococcaceae UCG-014* | 5.28E-04 | 0.04420951 | 0.003691769 | 0.046666819 |
|  | Cluster_27 | *-* | *-* | *-* | unknown genus | 5.21E-04 | 0.04420951 | 0.010688717 | 0.028979749 |
| **Line** | **OTU** | **phylum** | **class** | **family** | **genus** | **p-values** | **FDR** | **Mean relative abundance** | |
| **(OTU number)** |  |  |  |  |  |  |  | **V-Ch** | **NV-Ch** |
| **B14-line (5 OTUs)** | Cluster_667 | *Firmicutes* | *Clostridia* | *Lachnospiraceae* | *Lachnospiraceae UCG-006* | 6.06E-04 | 0.03878438 | 3.77E-05 | 0.000734925 |
|  | Cluster_155 | *-* | *-* | *-* | *ASF356* | 8.45E-06 | 0.00270524 | 2.47E-05 | 0.001144244 |
|  | Cluster_62 | *-* | *-* | *-* | *-* | 2.99E-04 | 0.0245376 | 1.44E-02 | 0.008150243 |
|  | Cluster_25 | *-* | *-* | *-* | *Eisenbergiella* | 2.81E-04 | 0.0245376 | 5.63E-03 | 0.000918581 |
|  | Cluster_23 | *-* | *-* | *Ruminococcaceae* | *Negativibacillus* | 3.07E-04 | 0.0245376 | 7.37E-03 | 0.032608741 |
| **B15-line (9 OTUs)** | Cluster_151 | *Firmicutes* | *Clostridia* | *Lachnospiraceae* | *Tyzzerella 3* | 9.37E-05 | 0.0118375 | 1.50E-03 | 4.34E-04 |
|  | Cluster_265 | *-* | *-* | *-* | *-* | 8.85E-04 | 0.03728622 | 5.58E-04 | 1.46E-04 |
|  | Cluster_33 | *-* | *-* | *-* | unknown genus | 1.79E-04 | 0.01360514 | 1.71E-02 | 7.49E-03 |
|  | Cluster_39 | *-* | *-* | *Ruminococcaceae* | *Ruminococcaceae UCG-014* | 1.88E-05 | 0.0071222 | 1.00E-05 | 2.06E-02 |
|  | Cluster_152 | *-* | *-* | *-* | *-* | 1.36E-04 | 0.01291593 | 5.47E-05 | 1.91E-03 |
|  | Cluster_6 | *-* | *-* | *-* | *Faecalibacterium* | 7.99E-04 | 0.03728622 | 1.30E-05 | 7.08E-02 |
|  | Cluster_66 | *-* | *-* | *-* | unknown genus | 8.45E-05 | 0.0118375 | 6.27E-05 | 1.03E-02 |
|  | Cluster_174 | *-* | *-* | *-* | *Ruminiclostridium 9* | 8.22E-04 | 0.03728622 | 1.97E-03 | 9.50E-05 |
|  | Cluster_553 | *-* | *-* | *-* | *DTU089* | 3.74E-04 | 0.02360288 | 2.58E-04 | 7.27E-05 |
| **B19-line (22 OTUs)** | Cluster_151 | *Firmicutes* | *Clostridia* | *Lachnospiraceae* | *Tyzzerella 3* | 5.71E-04 | 0.02022825 | 6.69E-04 | 2.26E-04 |
|  | Cluster_106 | *-* | *-* | *-* | *Lachnospiraceae UCG-008* | 2.35E-04 | 0.01227608 | 2.28E-03 | 3.74E-04 |
|  | Cluster_196 | *-* | *-* | *-* | *Agathobacter* | 2.80E-03 | 0.04961069 | 1.24E-03 | 3.87E-05 |
|  | Cluster_57 | *-* | *-* | *-* | *Ruminococcus torques group* | 2.78E-03 | 0.04961069 | 8.25E-03 | 2.49E-03 |
|  | Cluster_537 | *-* | *-* | *-* | unknown genus | 1.72E-03 | 0.03955873 | 4.42E-04 | 7.99E-05 |
|  | Cluster_403 | *-* | *-* | *-* | *Eisenbergiella* | 2.23E-03 | 0.0433917 | 5.46E-04 | 5.10E-05 |
|  | Cluster_590 | *-* | *-* | *-* | *-* | 1.01E-03 | 0.02633729 | 2.13E-04 | 2.86E-05 |
|  | Cluster_64 | *-* | *-* | *-* | *Blautia* | 2.12E-03 | 0.0433917 | 7.02E-03 | 2.73E-04 |
|  | Cluster_205 | *-* | *-* | *-* | *-* | 3.22E-04 | 0.01396602 | 7.18E-05 | 8.91E-04 |
|  | Cluster_161 | *-* | *-* | *-* | *Lachnoclostridium* | 8.20E-04 | 0.02460358 | 1.02E-04 | 7.78E-04 |
|  | Cluster_112 | *-* | *-* | *-* | unknown genus | 3.54E-07 | 0.00013799 | 2.45E-05 | 3.69E-03 |
|  | Cluster_39 | *-* | *-* | *Ruminococcaceae* | *Ruminococcaceae UCG-014* | 2.13E-06 | 0.00041594 | 8.67E-05 | 1.23E-02 |
|  | Cluster_125 | *-* | *-* | *-* | *-* | 3.17E-05 | 0.00377052 | 4.93E-05 | 5.57E-03 |
|  | Cluster_120 | *-* | *-* | *-* | *-* | 1.86E-04 | 0.01208994 | 2.61E-04 | 1.48E-03 |
|  | Cluster_17 | *-* | *-* | *-* | *-* | 2.22E-03 | 0.0433917 | 2.05E-03 | 5.83E-03 |
|  | Cluster_6 | *-* | *-* | *-* | *Faecalibacterium* | 2.52E-04 | 0.01227608 | 1.23E-05 | 7.56E-02 |
|  | Cluster_148 | *-* | *-* | *-* | *Candidatus Soleaferrea* | 4.14E-04 | 0.01615027 | 2.71E-04 | 2.53E-03 |
|  | Cluster_137 | *-* | *-* | *-* | unknown genus | 9.35E-04 | 0.02603515 | 1.68E-04 | 4.00E-03 |
|  | Cluster_66 | *-* | *-* | *-* | unknown genus | 1.59E-04 | 0.01208994 | 1.37E-04 | 4.51E-03 |
|  | Cluster_159 | *-* | *-* | *-* | *Ruminiclostridium 5* | 7.82E-04 | 0.02460358 | 1.55E-03 | 2.65E-04 |
|  | Cluster_174 | *-* | *-* | *-* | *Ruminiclostridium 9* | 1.72E-03 | 0.03955873 | 5.25E-04 | 1.06E-04 |
|  | Cluster_121 | *-* | *-* | *Clostridiales vadinBB60 group* | unknown genus | 3.87E-05 | 0.00377052 | 1.25E-04 | 1.12E-03 |
| **B21-line (9 OTUs)** | Cluster_148 | *Firmicutes* | *Clostridia* | *Ruminococcaceae* | *Candidatus Soleaferrea* | 1.09E-04 | 0.01370441 | 1.29E-04 | 2.01E-03 |
|  | Cluster_52 | *-* | *-* | *-* | *Ruminococcaceae UCG-014* | 2.65E-04 | 0.02000115 | 2.43E-04 | 3.89E-02 |
|  | Cluster_125 | *-* | *-* | *-* | *-* | 4.21E-04 | 0.02654502 | 3.29E-05 | 2.50E-03 |
|  | Cluster_152 | *-* | *-* | *-* | *-* | 5.80E-04 | 0.02942065 | 3.50E-04 | 2.13E-03 |
|  | Cluster_164 | *-* | *-* | *-* | *-* | 6.23E-04 | 0.02942065 | 1.91E-03 | 2.51E-04 |
|  | Cluster_205 | *-* | *-* | *Lachnospiraceae* | *Blautia* | 2.24E-04 | 0.02000115 | 1.61E-04 | 1.96E-03 |
|  | Cluster_64 | *-* | *-* | *-* | *-* | 3.80E-06 | 0.00143567 | 4.21E-03 | 2.41E-04 |
|  | Cluster_57 | *-* | *-* | *-* | *Ruminococcus torques group* | 8.45E-06 | 0.00159738 | 8.28E-03 | 9.81E-04 |
|  | Cluster_537 | *-* | *-* | *-* | unknown genus | 1.17E-03 | 0.04924893 | 4.98E-04 | 2.31E-05 |

**Supplementary Table S5:** Alpha diversity indices measured on the caecal microbial community of 9-weeks-old hens infected with IBV.

| Sample | Richness | Chao1 | Shannon | InvSimpson |
| --- | --- | --- | --- | --- |
| Ca10 | 374 | 398 | 4,22 | 33,03 |
| Ca11 | 385 | 410 | 4,27 | 35,29 |
| Ca13 | 371 | 401 | 4,21 | 30,87 |
| Ca14 | 406 | 427 | 4,37 | 42,52 |
| Ca15 | 321 | 337 | 3,84 | 19,90 |
| Ca16 | 340 | 361 | 4,07 | 28,07 |
| Ca17 | 329 | 353 | 3,90 | 18,34 |
| Ca18 | 382 | 401 | 4,27 | 33,23 |
| Ca19 | 402 | 419 | 4,30 | 36,22 |
| Ca1 | 373 | 387 | 4,09 | 25,68 |
| Ca20 | 390 | 406 | 4,24 | 35,98 |
| Ca21 | 364 | 377 | 4,01 | 17,24 |
| Ca22 | 341 | 366 | 3,61 | 13,27 |
| Ca23 | 372 | 396 | 4,14 | 22,54 |
| Ca24 | 363 | 389 | 4,12 | 32,70 |
| Ca25 | 306 | 333 | 3,67 | 15,65 |
| Ca26 | 368 | 401 | 4,32 | 40,04 |
| Ca27 | 293 | 358 | 3,97 | 31,08 |
| Ca28 | 405 | 418 | 4,43 | 40,11 |
| Ca29 | 291 | 333 | 3,68 | 19,44 |
| Ca2 | 358 | 388 | 3,94 | 14,01 |
| Ca30 | 309 | 319 | 3,83 | 22,22 |
| Ca31 | 327 | 359 | 3,89 | 25,96 |
| Ca32 | 394 | 420 | 4,23 | 33,20 |
| Ca33 | 341 | 379 | 3,92 | 23,92 |
| Ca34 | 318 | 332 | 3,89 | 25,21 |
| Ca35 | 249 | 264 | 3,16 | 9,41 |
| Ca36 | 282 | 304 | 3,68 | 18,54 |
| Ca37 | 352 | 374 | 4,15 | 27,22 |
| Ca38 | 324 | 344 | 4,01 | 31,40 |
| Ca39 | 352 | 381 | 4,17 | 35,41 |
| Ca3 | 272 | 286 | 3,08 | 5,98 |
| Ca40 | 283 | 303 | 3,71 | 20,70 |
| Ca41 | 380 | 392 | 4,14 | 30,39 |
| Ca42 | 364 | 388 | 4,22 | 26,96 |
| Ca43 | 348 | 360 | 3,99 | 26,28 |
| Ca44 | 398 | 442 | 4,14 | 30,01 |
| Ca45 | 377 | 391 | 4,21 | 28,35 |
| Ca46 | 355 | 373 | 4,30 | 35,81 |
| Ca47 | 349 | 381 | 4,17 | 29,09 |
| Ca48 | 353 | 374 | 4,15 | 33,81 |
| Ca49 | 340 | 379 | 3,71 | 15,71 |
| Ca4 | 392 | 412 | 4,12 | 26,79 |
| Ca50 | 350 | 393 | 4,16 | 35,01 |
| Ca51 | 367 | 396 | 4,07 | 29,93 |
| Ca52 | 326 | 346 | 3,52 | 7,67 |
| Sample | Richness | Chao1 | Shannon | InvSimpson |
| Ca53 | 359 | 375 | 4,02 | 27,59 |
| Ca54 | 354 | 378 | 3,60 | 13,84 |
| Ca55 | 290 | 308 | 3,02 | 5,57 |
| Ca56 | 385 | 396 | 4,14 | 30,60 |
| Ca57 | 325 | 353 | 3,91 | 24,91 |
| Ca58 | 282 | 315 | 3,46 | 13,12 |
| Ca59 | 325 | 331 | 3,76 | 19,22 |
| Ca5 | 378 | 391 | 4,17 | 26,37 |
| Ca60 | 351 | 369 | 3,74 | 16,36 |
| Ca61 | 351 | 378 | 4,04 | 27,25 |
| Ca62 | 352 | 374 | 3,74 | 12,98 |
| Ca63 | 311 | 358 | 3,67 | 17,34 |
| Ca64 | 338 | 368 | 3,52 | 9,97 |
| Ca65 | 341 | 364 | 4,17 | 29,91 |
| Ca66 | 291 | 320 | 3,37 | 10,62 |
| Ca67 | 383 | 410 | 4,18 | 31,15 |
| Ca68 | 343 | 356 | 3,96 | 23,20 |
| Ca69 | 364 | 381 | 3,88 | 14,15 |
| Ca6 | 340 | 367 | 3,87 | 17,33 |
| Ca70 | 360 | 399 | 3,95 | 20,07 |
| Ca71 | 323 | 345 | 3,94 | 23,57 |
| Ca72 | 382 | 394 | 4,26 | 35,95 |
| Ca73 | 350 | 365 | 3,91 | 18,11 |
| Ca74 | 244 | 260 | 3,69 | 18,32 |
| Ca75 | 378 | 399 | 4,29 | 37,11 |
| Ca76 | 336 | 357 | 3,82 | 19,65 |
| Ca77 | 298 | 309 | 3,24 | 7,87 |
| Ca78 | 355 | 380 | 3,76 | 17,87 |
| Ca79 | 335 | 362 | 3,69 | 17,03 |
| Ca80 | 291 | 306 | 3,48 | 13,18 |
| Ca81 | 302 | 319 | 3,63 | 17,04 |
| Ca82 | 300 | 315 | 3,54 | 13,96 |
| Ca83 | 304 | 324 | 3,29 | 11,04 |
| Ca84 | 297 | 334 | 3,61 | 17,60 |
| Ca85 | 332 | 348 | 3,83 | 20,06 |
| Ca86 | 360 | 374 | 3,92 | 18,02 |
| Ca87 | 159 | 170 | 3,66 | 25,54 |
| Ca88 | 303 | 325 | 3,64 | 15,49 |
| Ca89 | 258 | 274 | 3,54 | 14,21 |
| Ca90 | 229 | 240 | 3,56 | 17,50 |
| Ca91 | 350 | 361 | 3,98 | 26,40 |
| Ca92 | 241 | 262 | 3,57 | 14,67 |
| Ca93 | 367 | 383 | 4,03 | 27,05 |
| Ca94 | 252 | 264 | 3,52 | 14,20 |
| Ca95 | 270 | 282 | 3,58 | 15,06 |
| Ca96 | 340 | 365 | 3,83 | 17,74 |
| Ca9 | 361 | 374 | 4,17 | 31,78 |

**Supplementary Table S7:** Number of OTUs per genus with significantly different relative abundance between couple of lines. Only genera that displayed at least 2 OTUs differing between at least two couple of line are shown.

|  |  | **B12-line** | **B14-line** | **B19-line** | **B15-line** | **B2-line** | **B21-line** |
| --- | --- | --- | --- | --- | --- | --- | --- |
| **Genera with more abundant OTU in B14 line** | *Ruminococcus torques group* | 5 | - | 6 | 7 | 8 | 13 |
|  | *Escherichia-Shigella* | 0 | - | 2 | 0 | 3 | 3 |
|  | *Tyzzerella 3* | 2 | - | 3 | 0 | 1 | 0 |
|  | *Fusicatenibacter* | 0 | - | 2 | 0 | 2 | 2 |
|  | *Eisenbergiella* | 0 | - | 1 | 2 | 3 | 2 |
|  | *Sellimonas* | 0 | - | 2 | 0 | 3 | 3 |
|  | *Lachnoclostridium* | 0 | - | 2 | 2 | 1 | 1 |
| **Genera with less abundant OTU in B14 line** | *Ruminococcaceae UCG-014* | 3 | - | 6 | 5 | 11 | 11 |
|  | *Ruminococcus torques group* | 0 | - | 1 | 1 | 2 | 2 |
|  | *Ruminiclostridium 5* | 0 | - | 5 | 1 | 4 | 2 |
|  | *GCA-900066575* | 0 | - | 1 | 2 | 0 | 7 |
|  | *DTU089* | 0 | - | 1 | 2 | 0 | 2 |
|  | *Lachnospiraceae UCG-008* | 0 | - | 0 | 3 | 2 | 2 |
|  | *Lachnospiraceae NK4A136* | 0 | - | 2 | 0 | 2 | 1 |
| **Genera with more abundant OTU in B21 line** | *Ruminococcaceae UCG-014* | 5 | 11 | 0 | 0 | 0 | - |
|  | *Lachnoclostridium* | 3 | 2 | 0 | 0 | 0 | - |
|  | *Lachnospiraceae UCG-008* | 4 | 2 | 0 | 0 | 0 | - |
|  | *GCA-900066575* | 4 | 7 | 0 | 0 | 0 | - |
|  | *DTU089* | 2 | 2 | 0 | 0 | 0 | - |
|  | *Ruminococcus torques group* | 3 | 2 | 0 | 0 | 0 | - |
| **Genera with less abundant OTU in B21 line** | *Oscillibacter* | 4 | 2 | 0 | 0 | 0 | - |
|  | *Flavonifractor* | 5 | 5 | 0 | 0 | 0 | - |
|  | *Escherichia-Shigella* | 4 | 3 | 0 | 0 | 0 | - |
|  | *Ruminococcus 1* | 2 | 2 | 0 | 0 | 0 | - |

**Supplementary Table S8:** Pair-wise comparison of beta-diversity similarity between each couple of line, according to the vaccine status in IBV challenged laying hens. Results of the Adonis testing for the difference of Bray-Curtis distance between each couple of lines. The table indicates the p-value of the Adonis testing for the difference of Bray-Curtis distance between each couple of lines in the vaccinated challenged(V-Ch) and non vaccinated challenged (NV-Ch) groups separately. The p-values are highlighted in bold characters when significant (* <0.05, ** <0.01, and *** <0.001).

| **Vaccine status** |  | **B12-line** | **B14-line** | **B19-line** | **B15-line** | **B2-line** |
| --- | --- | --- | --- | --- | --- | --- |
| **V-Ch** | **B14-line** | **0.0252 *** |  |  |  |  |
|  | **B19-line** | 0.1132 | **0.002 **** |  |  |  |
|  | **B15-line** | 0.1371 | **0.0007 ***** | 0.5216 |  |  |
|  | **B2-line** | **0.0027 **** | **0.0003 ***** | 0.2476 | 0.056 |  |
|  | **B21-line** | **0.0299 *** | **0.0028 **** | 0.5428 | 0.619 | 0.2331 |
| **NV-Ch** | **B14-line** | **0.0006 ***** |  |  |  |  |
|  | **B19-line** | **0.0105 *** | **0.0004 ***** |  |  |  |
|  | **B15-line** | 0.1105 | **0.003 **** | 0.2133 |  |  |
|  | **B2-line** | 0.1325 | **0.0018 **** | 0.2363 | 0.0609 |  |
|  | **B21-line** | **0.0004 ***** | **0.0001 ***** | **0.0054 **** | **0.0024 **** | **0.0008 ***** |

**Supplementary Table S9:** Analysis of β-diversity on caecal microbiota by Adonis test. Association between the microbiota and the concomitant immune phenotype are described through the p-value of Adonis test based on Bray-Curtis distance matrix and each immune parameter level. The associations are described in the total population, and in each line separately. When significant, the p-value of the effect is highlighted in bold characters (*p<0.05, **<0.01, ***≤0.001).

| **Immune phenotype** | **Total population** |  | **B12-line** | **B14-line** | **B19-line** | **B15-line** | **B2-line** | **B21-line** |
| --- | --- | --- | --- | --- | --- | --- | --- | --- |
| Thrombocytes | 0.7875 |  | 0.6747 | 0.4885 | 0.4817 | 0.4642 | 0.3854 | 0.9116 |
| Heterophils | 0.2127 |  | 0.5187 | 0.7261 | 0.1503 | 0.6782 | 0.3361 | 0.3929 |
| **Monocytes** | 0.0652 |  | 0.3261 | 0.3319 | 0.167 | 0.7232 | 0.3555 | 0.3921 |
| B cells | 0.948 |  | 0.3093 | 0.2768 | 0.8219 | 0.654 | 0.5813 | 0.4106 |
| TCR _ϒδ+_ T cells | 0.5279 |  | 0.3873 | 0.7943 | 0.4657 | 0.2256 | 0.2653 | 0.6562 |
| **TCR1+CD8+ δϒ T cells** | 0.9901 |  | 0.5803 | 0.7014 | 0.6564 | 0.2193 | 0.7113 | 0.0887 |
| TCR1+CD8- δϒ T cells | 0.2726 |  | 0.2715 | 0.6636 | 0.485 | 0.1653 | 0.1536 | 0.6557 |
| (ab) T cells | 0.9281 |  | 0.5714 | 0.7549 | 0.4327 | 0.7187 | 0.6401 | 0.304 |
| **TCR1 CD4+ (ab) T cells** | 0.7792 |  | 0.6046 | 0.5536 | 0.2497 | 0.1578 | 0.8836 | 0.3738 |
| **TCR1-CD4+ (ab) T cells** | 0.1922 |  | 0.4512 | 0.9586 | 0.1688 | 0.1664 | 0.6701 | 0.6124 |
| TCR1-CD4+CD25+ (ab) T cells | 0.5448 |  | 0.7578 | 0.9323 | **0.0123 *** | **0.0187 *** | 0.2607 | 0.7122 |
| **CD45 expression on thrombocytes** | 0.2158 |  | 0.7907 | 0.3087 | 0.1369 | 0.8339 | 0.5766 | 0.4162 |
| **CD45 expression on heterophils** | 0.2911 |  | 0.3954 | 0.7948 | 0.5906 | **0.0176 *** | 0.9433 | 0.4321 |
| MRC1L-B expression on monocytes | 0.5999 |  | 0.1924 | 0.3859 | 0.1934 | 0.7267 | 0.2807 | 0.7111 |
| **CD45 expression on monocytes** | **0.0303 *** |  | 0.1889 | 0.1887 | 0.099 | 0.9327 | 0.075 | **0.0191 *** |
| MHCII expression on monocytes | 0.4604 |  | 0.3027 | 0.0729 | 0.6507 | 0.9426 | 0.3164 | 0.6031 |
| **BU1 expression on B cells** | **0.0116 *** |  | 0.2249 | 0.0754 | **0.0116 *** | 0.4018 | 0.3529 | **0.0272 *** |
| **IgM on B cells** | **0.0159 *** |  | 0.0906 | 0.4423 | 0.7193 | 0.8499 | 0.5344 | 0.0985 |
| **TCR_ϒδ_ expression on δϒ T cells** | **0.0005 ***** |  | 0.1574 | **0.0157 *** | 0.6884 | **0.0354 *** | 0.1957 | **0.0079 **** |
| CD8β expression on CD8αβ+ TCR δϒ cells | 0.4938 |  | 0.6617 | 0.8826 | 0.0876 | 0.5558 | 0.8397 | 0.3429 |
| CD8β expression on CD8αβ+ T cells | 0.3367 |  | 0.3527 | 0.4672 | 0.0943 | 0.14 | 0.9791 | 0.8766 |
| **CD4 expression on CD4+ T cells** | 0.3352 |  | 0.3691 | 0.1203 | 0.3149 | 0.1111 | 0.5588 | 0.122 |
| CD4 expression on CD4+ CD25+ T cells | 0.3052 |  | 0.31 | 0.7042 | 0.7025 | 0.1136 | 0.818 | 0.7171 |
| CD25 expression on CD4+ CD25+ T cells | 0.7377 |  | 0.6318 | 0.6986 | 0.3999 | 0.2565 | 0.9508 | 0.8431 |
| MHCII expression on B cells | 0.9944 |  | 0.1637 | 0.1839 | 0.1972 | 0.7596 | 0.891 | 0.3862 |
| IBV viral load (Log) | 0.4865 |  | 0.486 | 0.6782 | 0.5887 | 0.2484 | 0.8333 | 0.8556 |
